# Atomistic Picture of Opening–Closing Dynamics of DNA Holliday Junction Obtained by Molecular Simulations

**DOI:** 10.1021/acs.jcim.3c00358

**Published:** 2023-04-26

**Authors:** Zhengyue Zhang, Jiří Šponer, Giovanni Bussi, Vojtěch Mlýnský, Petr Šulc, Chad R. Simmons, Nicholas Stephanopoulos, Miroslav Krepl

**Affiliations:** † Institute of Biophysics of the Czech Academy of Sciences, Královopolská 135, 612 00 Brno, Czech Republic; ‡ CEITEC—Central European Institute of Technology, Masaryk University, Kamenice 5, 625 00 Brno, Czech Republic; § National Center for Biomolecular Research, Faculty of Science, Masaryk University, Kamenice 5, 625 00 Brno, Czech Republic; ∥ Scuola Internazionale Superiore di Studi Avanzati (SISSA), via Bonomea 265, 34136 Trieste, Italy; ⊥ Biodesign Center for Molecular Design and Biomimetics, Arizona State University, 1001 S. McAllister Ave, Tempe, 85287 Arizona, United States; # Regional Centre of Advanced Technologies and Materials, Czech Advanced Technology and Research Institute (CATRIN), Palacky University Olomouc, Slechtitelu 241/27, 783 71 Olomouc, Czech Republic

## Abstract

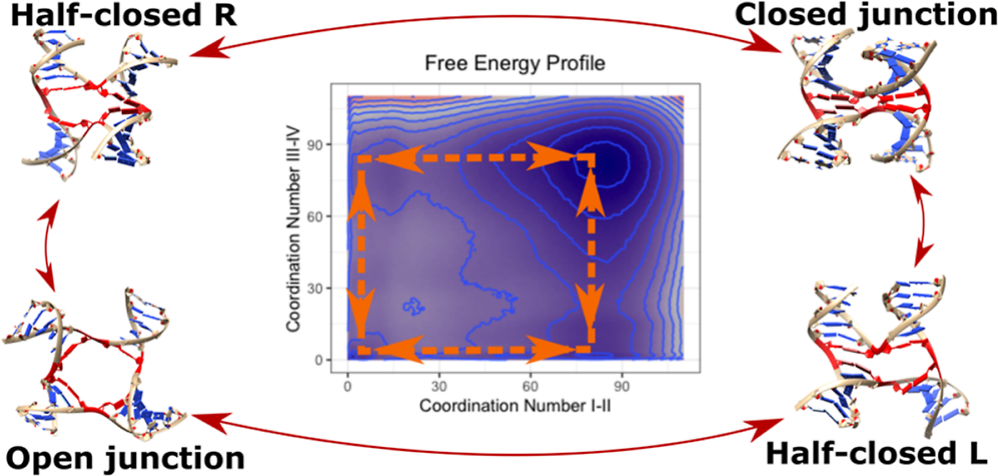

Holliday junction (HJ) is a noncanonical four-way DNA structure with a prominent role in DNA repair, recombination, and DNA nanotechnology. By rearranging its four arms, HJ can adopt either closed or open state. With enzymes typically recognizing only a single state, acquiring detailed knowledge of the rearrangement process is an important step toward fully understanding the biological function of HJs. Here, we carried out standard all-atom molecular dynamics (MD) simulations of the spontaneous opening–closing transitions, which revealed complex conformational transitions of HJs with an involvement of previously unconsidered “half-closed” intermediates. Detailed free-energy landscapes of the transitions were obtained by sophisticated enhanced sampling simulations. Because the force field overstabilizes the closed conformation of HJs, we developed a system-specific modification which for the first time allows the observation of spontaneous opening–closing HJ transitions in unbiased MD simulations and opens the possibilities for more accurate HJ computational studies of biological processes and nanomaterials.

## Introduction

**Figure 1:**
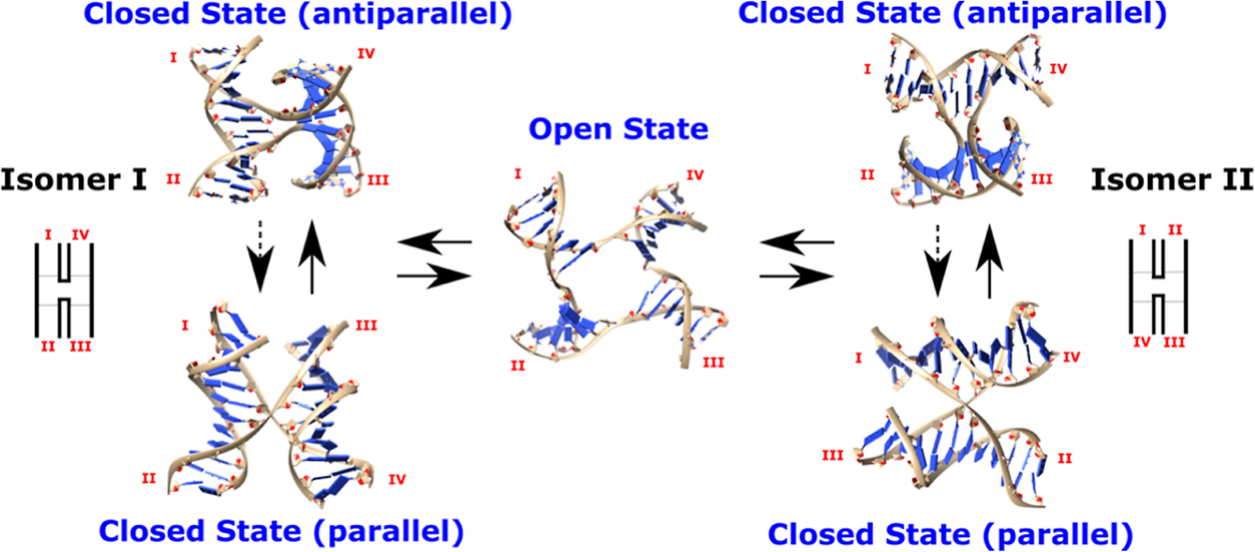
Conformational states of HJs and their transitions. The solid and dashed arrows refer to transition pathways with high and low probabilities, respectively, as shown by our simulations. Different arrangements of the helical arms are labeled and result in two HJ closed isomers—I and II. The transitions between them proceed *via* the open state.

Holliday junctions (HJs) are noncanonical DNA structures with four joined duplexes (Figure 1).^1,2^ The formation of HJs is the cornerstone of homologous recombination, yielding a motif prominent in DNA repair and meiosis.^1,3^ Accurate recognition of HJs by proteins ensures stable genetic information processing, while their ability for branch migration or base pair exchange in homologous DNA regions allows for genetic variety.^4−8^ The cross-like conformation of HJs is also a widely utilized tool in nanomaterial science where it is the basic structural unit for building DNA nanostructures such as DNA crystals, tiles, and origamis.^9−15^

An intriguing structural aspect of HJs in solution is their conformational transitions between the stacked (closed) and open states depending on ionic strength.^16−19^ The transitions enable sequence-specific junction cleavage by resolvases and permit branch migration in the open state.^4−6,17,18,20^ Basic characteristics of HJ opening–closing transitions were obtained from the studies of free HJs under different ionic environments. These studies revealed that the open state is prevalent at low monovalent cation concentration (<40 mM), while the closed state becomes preferred at higher monovalent concentrations (>150 mM) and in the presence of divalent cations.^17,18,21,22^ Although the factors governing the balance between open and closed HJs have been elucidated,^17,18,22,23^ the atomistic details of the opening–closing process remain elusive due to the fast dynamics of the process and the resolution limitations.^16,17,24^ The commonly assumed two-state model for opening and closing ignores the structural complexity and conformational flexibility of HJs. For example, there are two possible stacking patterns among the HJ’s arms which comprise the closed conformation, resulting in two isomers (I and II) which can interexchange *via* the open state (Figure 1).^25−27^ Interhelical dynamics of the closed forms, including transitions between the antiparallel and parallel conformations, further complicate the conformational landscape of HJs (Figure 1).^2,28^

Molecular dynamics (MD) simulation is a powerful technique for studying biomolecular movement, with a spatial and time resolution unrivalled by existing experimental methods.^29−31^ MD has been successfully applied to study conformational flexibility of free HJs in solution,^9,14,32−34^ as well as complexed with proteins.^35,36^ Some of the MD studies, however, reported force-field issues,^34,35,37^ most notably the inability to maintain the open form of HJ.^33,35^ Although the HJ should predominantly exist in the open form under low-salt conditions,^17,18,21,22,24,38^ the open form HJ promptly and permanently closes in simulations.^33,35^ Likewise, the junction never spontaneously opens when starting the simulation from the closed conformation. Potential issues leading to the inability of the force field to describe open HJs are overestimated phosphate–cation interactions that screen the electrostatic repulsions at the junction branch point,^37^ excessive base–base stacking,^39,40^ and overestimated sugar–sugar van der Waals (vdW) interactions.^37^ Although experimental studies indicated relatively infrequent opening–closing dynamics of the HJ compared to the typical timescales of MD,^16,24,38^ the complete instability and inaccessibility of the open state in numerous MD simulations is an obvious force-field issue and represents a bottleneck for, e.g., simulation studies of sequence-dependent stability of different HJs. More importantly, it prevents studies of the transitions between isomers I and II in standard MD simulations as these inevitably involve the open state as the intermediate (Figure 1). The complete absence of the open form in simulations also prevents studies of HJ conformational dynamics at different ion concentrations. Notably, this information is difficult to get by experiments and therefore scarce in the literature.^17,21,41^ Were a reasonable description of the open form HJ achieved by the force field, the simulation studies could significantly expand our knowledge about HJ dynamics.

**Figure 2:**
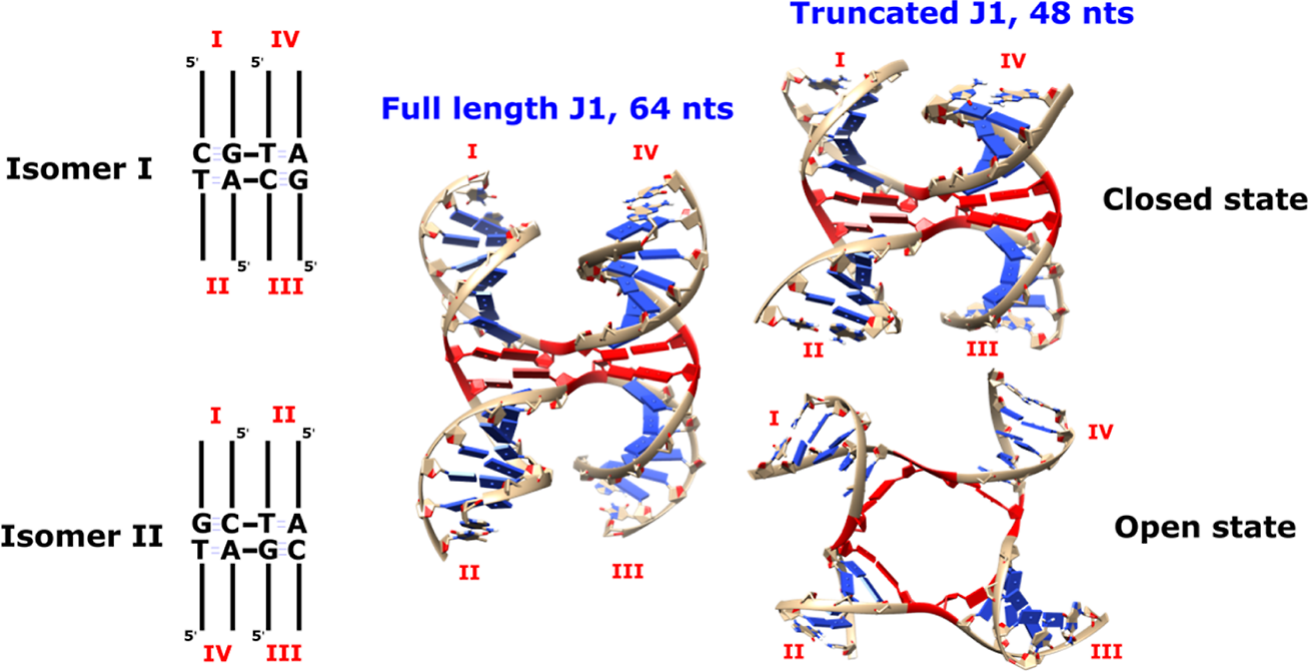
Models of HJ 1 (J1) used in the simulations. Schemes of the two isomers of J1 with the stems (I–IV) labeled (left). The “full-length” HJ structure contains 64 nucleotides (middle). In most standard MD simulations, we used slightly truncated J1 system with 48 nucleotides (right) to increase the speed of the simulations, while all enhanced sampling simulations were executed with the “full-length” HJ. The branch point nucleotides are in red. Both closed and open states are shown for the truncated system.

In this work, we use MD simulations to explore the opening–closing transitions of the HJ type 1 (J1) sequence, which is the most commonly studied immobile HJ (Figure 2).^42^ We performed a multitude of standard MD simulations followed by the enhanced sampling simulations that combined Well-tempered MetaDynamics with Hamiltonian Replica Exchange (WT-MetaD-HREX).^43,44^ The standard simulations allow us to obtain an unbiased qualitative picture of the transitions, while enhanced sampling can be used to more thoroughly explore the transitional pathways and to obtain the corresponding free-energy profiles. In addition to J1, we also performed several simulations of J2 and J13, which were reported to have different isomerization stability and crystallization capability compared with J1.^14,26^ The two additional junctions are used to evaluate the potential sequence effects on the HJ dynamics. Our simulations reveal complex transitions between the closed and open HJ states and estimate their relative stabilities and the effects of the DNA sequence. This work is the first to comprehensively describe the conformational landscape of the HJ transitions and characterize a previously unknown “half-closed” intermediate state through which the vast majority of the transitions proceed.

In order to study the HJ opening–closing dynamics, we had to make a very substantial modification of the OL15^45−47^ DNA AMBER force field, namely, large-scale weakening of the vdW interactions at the branch point nucleotides. This is because the standard AMBER nonbonded force-field terms^48^ dramatically overstabilize the HJ’s closed state, inhibiting any opening events and preferring the closed state even at ion concentrations where the open form should be greatly preferred. The modification is based on the nonbonded fix (NBfix) approach, which is a change of the standard vdW combination rules (Lennard-Jones parameters) for selected atom pairs that allows the tuning of intermolecular interactions.^37^ As discussed below, the overstabilization of the closed HJ conformation by the force field is so large and complicated that it is unlikely to be correctable by any general reparameterization as this would be detrimental for other systems. Therefore, we resort to a system-specific correction. Furthermore, as an auxiliary modification, we applied the established CUfix NBfix parameters developed by Aksimentiev et al.^37^ to reduce the screening of the phosphate–phosphate repulsion by the cation atmosphere.

## Results

In this work, we use MD simulations to explore the opening–closing transitions of HJs. Since spontaneous transitions are not observable with the standard force field due to large overstabilization of the closed state, we develop a system-specific variant of the AMBER OL15 force field^45−47^ where we scale down pairwise Lennard-Jones (LJ) potentials for selected atoms of the branch point nucleotides. With this modification, spontaneous opening and closing transitions can be readily observed in standard MD simulations. We subsequently for the first time describe the “half-closed” states as the intermediates along the dominant pathways employed by the HJ during the opening–closing transitions. By performing the WT-MetaD-HREX simulations, we also detail the free-energy landscapes among different HJ conformations and validate the response of HJ dynamics to a range of ion concentrations in MD simulations.

### HJ Never Opens in MD Simulations Using the Standard Force Field, Regardless of the Ion Concentration

The HJ systems simulated with the standard AMBER force field with different concentrations of K^+^ ions never show any spontaneous opening events. This is consistent with an earlier MD simulation study performed on all 36 immobile HJs,^14^ as well as observations made by other groups.^37^ Previously, we demonstrated that only complete removal of all explicit ions leads to spontaneous HJ opening in simulations.^35^ Such simulation conditions are quite extreme and illustrate the severity of the imbalance. Furthermore, the junction rapidly closes again once the ions are reintroduced, confirming the overstabilization of the closed conformation.

**Figure 3:**
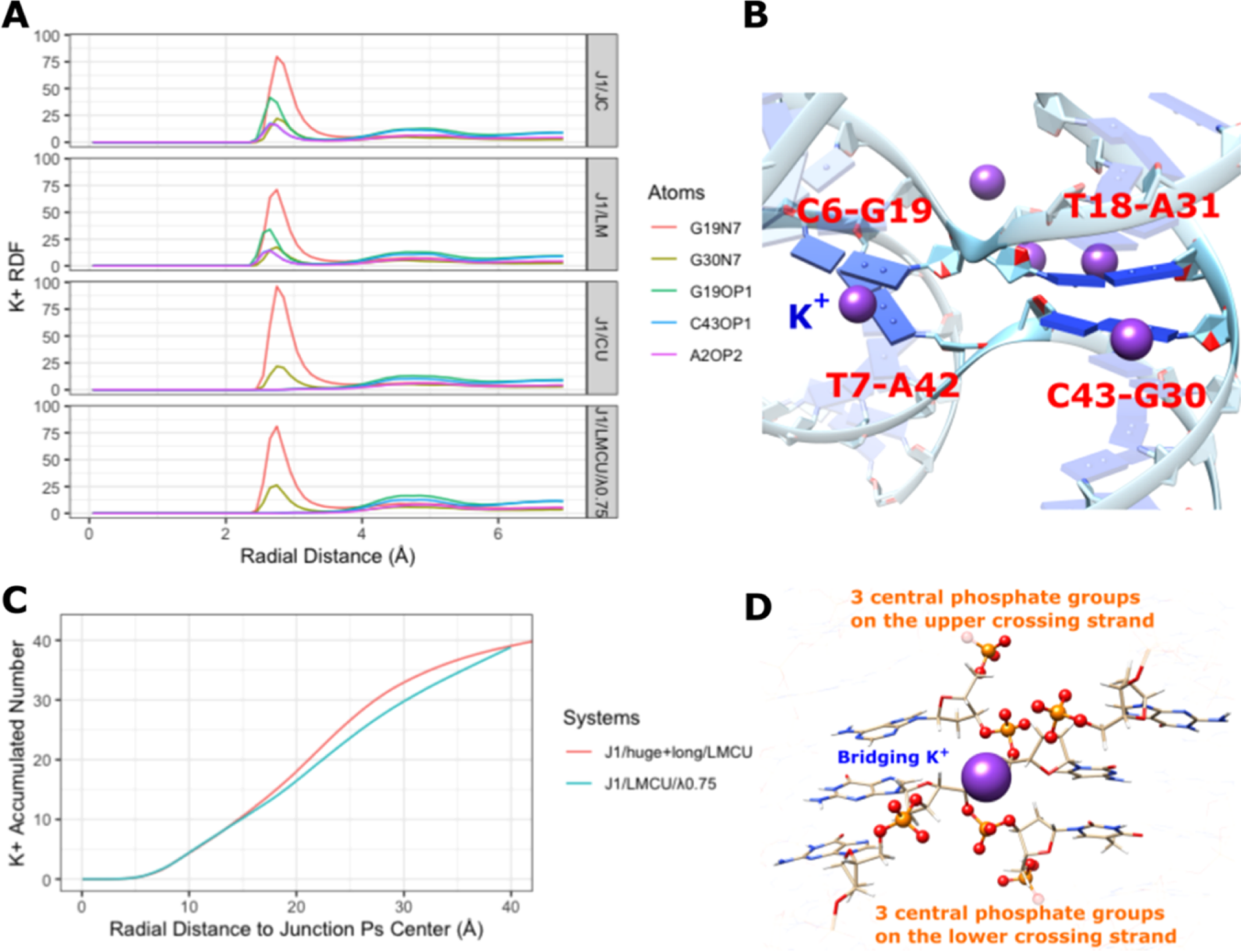
Potassium binding sites around the J1 junction. (A) Normalized radial distribution functions (RDFs) of K^+^ around selected junction atoms in simulations using different ion parameters and simulation conditions (see Table S1). (B) Besides the phosphate groups, the major and minor grooves at the center of the HJ also strongly bind K^+^. (C) The number of K^+^ ions around the center of the HJ (the geometric center of the two branch point phosphates on the crossing strands) in selected simulations with different K^+^ concentrations. (D) The three central phosphate groups on each crossing strand (ball-and-stick representation) are responsible for the large negative charge repulsion at the junction branch point, which is being screened by the potassium ion (violet sphere representation).

The binding of ions appears to be a possible culprit preventing the opening of HJs. Therefore, we first attempted to obtain a more realistic representation of the explicit ions. A well-known issue in nucleic acid simulations is the overestimation of the cation–phosphate interactions.^37,49^ To address this, we tested different ion parameters (Joung and Cheatham,^50^ JC and Li and Merz,^51^ LM; see Methods), as well as the earlier proposed CUfix modification^37^ which increases the optimal vdW distance between phosphate oxygens and cations. Indeed, the CUfix effectively reduced the contacts between ions and phosphate groups near the center of the junction, although the HJ still failed to open in simulations. In contrast, the choice of the ion parameters had only negligible effects (Table 1 and Figure 3A); therefore, we continued to use the LM parameters along with the CUfix in most of the subsequent simulations.

**Table 1:** Incidence of Ionic Bridging at the Branch Point Phosphates in HJ Simulations^a^

systems^b^	bridging (%)
JC	43.4
LM	43.4
JC + CU	27.9
LM/15K	38.0
LM/05K	30.8
LM/02K	20.8

a Ionic bridging is considered present when one or more potassium ions are located within 6 Å of any of the three central phosphate groups of both crossing strands simultaneously (Figure 3D).

b “JC” and “LM” indicate Joung and Cheatham^50^ and Li and Merz^51^ ion parameters, respectively. “CU” indicates that the CUfix modification^37^ was applied. “15K”, “05K”, and “02K” correspond to 0.15, 0.05, and 0.02 M K^+^ concentration, respectively. See Table S1 for details.

Another potential problem arises from the relatively small periodic boxes routinely utilized in MD simulations. Small boxes are used to keep the computational costs reasonable but can result into high effective solute concentrations. Consequently, the concentration of cations required for net-neutralization is also rather high (∼0.25 M, solute-box boundary distance = 12 Å), typically exceeding the physiological monovalent cation concentrations, as well as the ones applied in *in vitro* experiments.^16,18^ Another consequence is the lack of bulk solvent as we directly show that net-neutralizing conditions do not reproduce the correct ionic distribution due to the absence of co-ions (see the section below).^31,52,53^ In other words, the counterions are either located around the solute or they transition directly from one solute image to another without ever reaching a true bulk-like behavior. This significantly complicates the comparison with the experimental studies of HJ dynamics dependent on the cation concentration as the experimentally measured values and the calculated ones in simulations are not directly comparable (see below for further discussion).

In our simulations, we tried to mitigate the concentration problem either by setting the number of K^+^ ions to be less than required for net-neutralization (i.e., the subneutralizing cation conditions; see Methods) or by expanding the boxes (Table S1), thus reducing the simulation cation concentration at net-neutral conditions; however, none of these approaches led to spontaneous opening of the HJ. The opening events were not observable even at *c*(K^+^) = 0.02 M, contradicting the experiments which show that the open HJ enormously predominates at such ion concentration.^2,17,18,21,22,27,38,54^ A possible reason for this is that cations bind to major and minor grooves at the HJ branch point in addition to screening the repulsion between phosphates (Figure 3A,B).^14,55^ These ion sites appear to be unrelated to the opening–closing transitions, but they could contribute to the relatively high local ionic concentration which stabilizes the closed state. In other words, even small number of K^+^ still predominantly congregates around the center of the stacked junction and the local K^+^ concentration is high regardless of the bulk ionic conditions (Figure 3C). The accumulated cations then effectively screen the electrostatic repulsion between the crossing DNA strands by forming ionic bridges (Figure 3D). These ionic bridges could be excessively populated in the standard pair-additive force field due to, e.g., the lack of polarization and charge transfer effects.^31,56,57^ At the same time, use of the computationally demanding polarizable force fields is well out of the scope of this study where we aim to correct the molecular mechanics description of the HJ dynamics in pair-additive all-atom simulations. Roughly, the same amount of sampling would be necessary to confidently evaluate the performance of the polarizable force field in a system as complex as the HJ. Interestingly, if starting the simulation from the open HJ in the huge water box or subneutralizing condition systems, the junction remained open throughout the simulation which likely only reflects insufficient sampling under these ionic conditions.

### Scaling vdW Interactions of the Central Nucleotides Enables the Opening–Closing Dynamics of the HJ

Besides the cation-based screening of the electrostatic repulsion (see above), the vdW interactions of the nucleotides at the HJ center are also a significant factor stabilizing the closed form. The base/base stacking might be overstabilized in MD simulations,^39,58−60^ although the magnitude of this overstabilization is likely context-dependent and sensitive to the water model.^31^ The presently used OPC water model should actually weaken stacking compared to models such as TIP3P or SPC/E,^31^ which is the main reason we chose it. Even nonspecific sugar/sugar vdW interactions could be excessive in the standard force field.^37^ Thus, we decided to develop a HJ-specific force-field modification weakening the vdW interactions at the HJ center by scaling down the pairwise LJ potentials between atoms of the central nucleotides (see Methods and Supporting Information). Using a massive set of standard simulations, we empirically found a suitable scaling factor (λ) that leads to spontaneous closing and opening of HJ in standard MD simulations. The scaling elevates the potential energy basin corresponding to the closed state and thus reduces the lifetime of the closed state (increases the rate constant associated with the opening process), altering also the open/closed state ratio. Based on the standard simulations, we estimated that the ideal λ at which both J1 closing and opening occur is 0.75 and 0.625 with and without CUfix,^37^ respectively. Single-point vdW interaction energy calculations using the minimized experimental closed J1 structure reveal that the λ values of 0.75 and 0.625 weaken the vdW interaction energy by ∼12 and ∼17 kcal/mol in the closed state, respectively (Figure S1). Note that this is a simple single-point calculation which evaluates the vdW potential energy and is not equivalent with the free energy which will be discussed later in the paper.

Overall, the results confirm that although the behavior of ions around the central part of the junction indeed contributes to the overstabilization of the closed state, the main culprit is the excessive vdW interaction. As the use of CUfix allows milder LJ potential scaling, all of the subsequent simulations used CUfix. The above two λ values indicate that the effect of CUfix accounts for about half of the contribution of employing the λ = 0.75 scaling. The combination of CUfix and the λ = 0.75 scaling leads to the opening–closing transitions in both the truncated and full-length HJ simulations. Note that the suggested ideal λ we conventionally reported is the highest value of a small interval in which the spontaneous HJ transitions in both directions can be observed. The highest value in this interval was then chosen in order to minimize the potential side effects introduced by the scaling.

### Standard MD Simulations Qualitatively Define the Conformational Landscape of Opening–Closing Transitions of HJ

**Figure 4:**
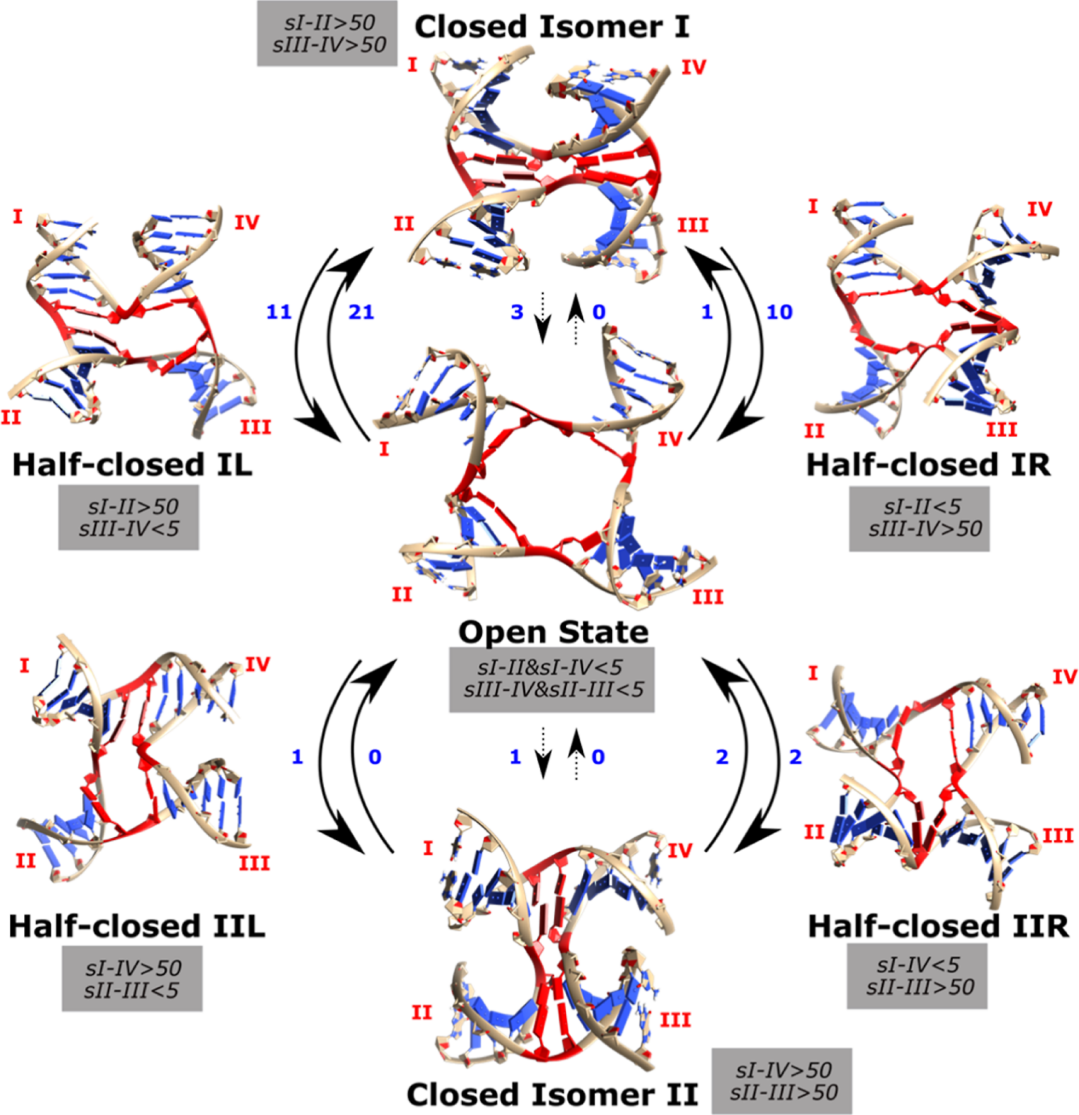
Conformational pathways of the opening–closing dynamics in standard MD simulations of the J1 junction. The clear majority of the opening–closing events proceeded *via* intermediate “half-closed” states IL/IR and IIL/IIR (full arrows), while direct transitions between the open and closed states were comparatively rare (dashed arrows). The blue numbers show the counts of the transitions between the indicated states seen in the reactive trajectories in the whole 181 μs-ensemble of standard J1 simulations, irrespective of the chosen solvent conditions, starting structures, or λ scaling factors. By reactive trajectories, we mean trajectory portions with complete transitions between two end states, i.e., from closed to open state or vice versa. The gray boxes show the substate definitions by the CVs used in the enhanced sampling calculations (see Methods for details).

As mentioned above, we were able to observe spontaneous closing and opening events of the HJ in multiple simulations, allowing us to detail the transition pathway. Notably, we observed previously unknown intermediate states for the opening–closing transitions in which only two helical arms of HJ are stacked, while the other two are separated. We term these states as “half-closed”. Depending on which HJ arms are stacked, two different half-closed states, “L” and “R”, exist along the transition pathway between each closed state isomer and the open state (Figure 4). The L and R intermediates have stem I stacked and unstacked, respectively, regardless of which stem stacks with stem I (Figure 2). Based on whether the intermediates lie on pathway toward isomer I or II, we then define four half-closed states, “IL”, “IR”, “IIL”, and “IIR” (Figure 4). The half-closed states are rather short-lived, with average observed lifetimes on nanosecond timescale. Nevertheless, these forms are significant as they participate in dominant pathways by which the HJ opens and closes in simulations. Direct transitions circumventing half-closed states were also sampled but only infrequently (∼7.7% over all the transitions). The simulations also revealed a clear preference for the isomer I-open state transitions of J1 to proceed by half-closed IL state. Namely, among 46 transitions observed in all MD simulations of J1 between closed state isomer I and open state, 32 of them passed through half-closed state IL while only 11 through half-closed state IR (Figure 4). It should be noted that there were also many unsuccessful transition attempts where the junction reached the half-closed state but then reverted back instead of completing the transition. A preference for specific half-closed intermediate is also observed for the isomer II-open state transitions, but we sampled far fewer of those than for isomer I. This was partly because we never started any simulations from the isomer II structure, but transitions starting from the open HJ could also reflect the experimentally known strong preference of J1 for isomer I.^16,27^ Although our standard MD simulations provided clear qualitative picture of the HJ opening–closing dynamics, there were still relatively few observed transitions, and their sampling is likely not converged. Therefore, we further explored the transitions by WT-MetaD-HREX simulations which provided us with enough data for free-energy analysis (see below). In addition, we also validated the existence of the half-closed intermediates in unmodified force field by pure WT-MetaD calculations, see Supporting Information.

### Closing–Opening Transition Pathways of HJ Detailed by Well-Tempered MetaDynamics with Hamiltonian Replica Exchange

**Figure 5:**
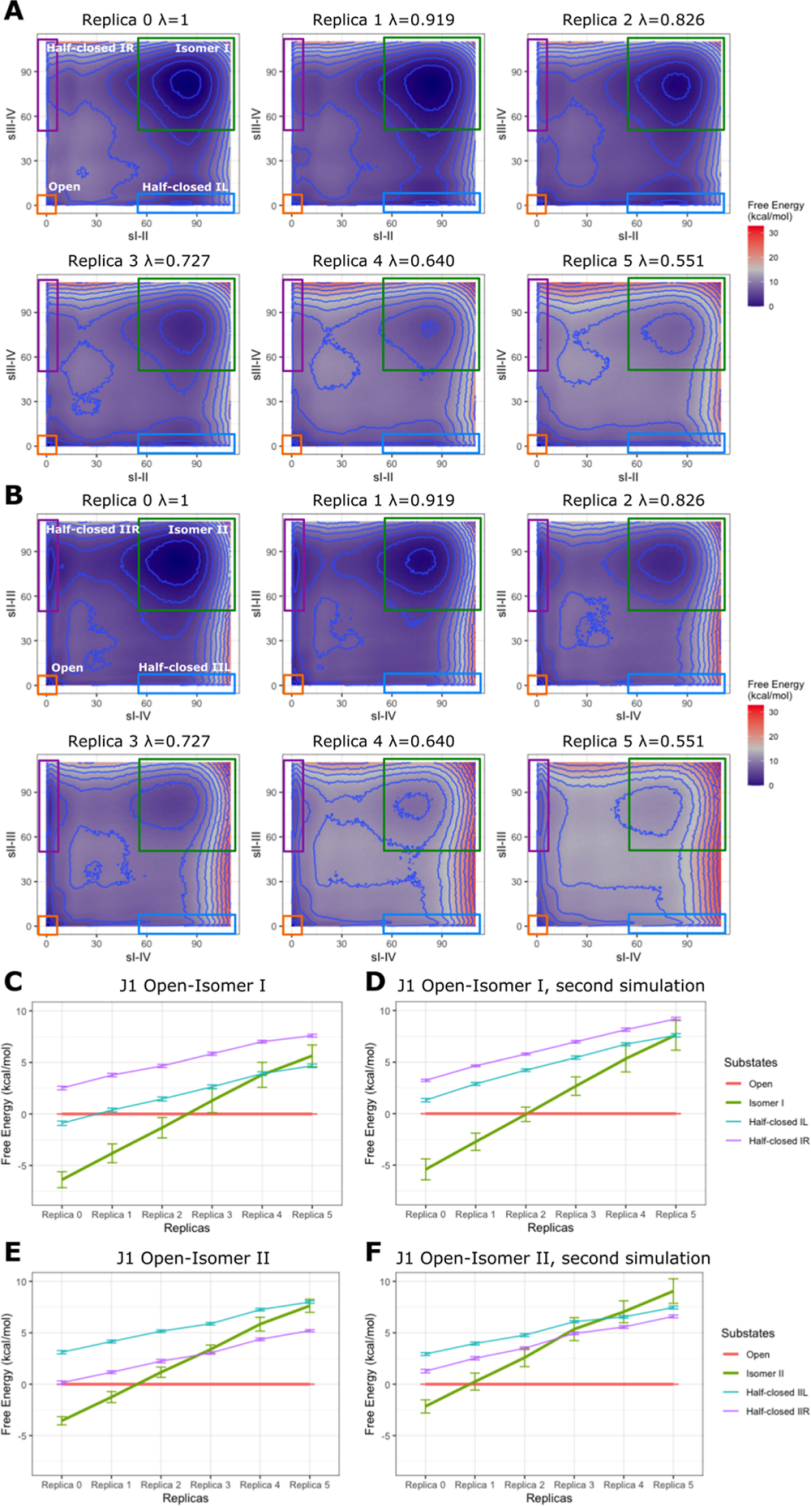
(A,B) Free-energy profiles of transitions between the open state and the closed states isomer I (A) and II (B) of J1 derived from the WT-MetaD-HREX simulations (see Methods for details). Data for the equivalent second runs are shown in Supporting Information. The 2D free-energy profiles are described by the sI-II and sIII-IV (A) and sI-IV and sII-III (B) CVs. The four rectangles indicate the approximate regions in the CVs’ space corresponding to the individual HJ states (labeled in replicas 0). The interval of the blue contour lines is 2 kcal/mol, and the free-energy minimum is set to 0. For the free-energy color gradient, we set 15 kcal/mol as the midpoint, namely, the gray color. By applying scaling (replicas with larger replica numbers), the open state becomes increasingly preferred over the closed state. (C–F) Free-energy difference of each state to the open state in the replica ladder (with different λs applied for the branch point nucleotides) in four WT-MetaD-HREX runs. The free-energy value of each state was calculated by integrating the probability density in regions defined by (A,B) with error bars calculated by bootstrapping analysis. We used the open state as reference in each replica, and thus, no error bar is set. See also Methods and Supporting Information for details. Note that in (A,B), the open state is localized in the very small area in bottom left corner, so the free-energy minimum is poorly visible at higher replicas; however, the (C–F) give the relative free energies of all states unambiguously.

We used the WT-MetaD-HREX method to boost the sampling of transitions for the J1 HJ. An important conceptual advantage of the WT-MetaD-HREX approach is that it uses a bias potential to flatten the distribution of selected collective variables (CVs), thus increasing conformational sampling. More specifically, in contrast to pure RE methods, it allows us to reconstruct the unbiased free-energy profiles for all replicas, i.e., for all λ values, irrespective of the magnitude of the free-energy differences among the different states.^61^ Two different WT-MetaD-HREX setups and CV combinations were applied to accelerate the transitions between the open state and either the closed state isomer I or II (Figures 5, S2). Each setup was run twice. Due to the computational demands, we could not perform a WT-MetaD-HREX simulation where we would simultaneously explore all three states (open state and closed state isomers I and II; see Figure 4) within one simulation run since the use of four CVs simultaneously in a four-dimensional MetaD setup would be required.

The WT-MetaD-HREX free-energy profiles nicely match the qualitative picture observed in the standard MD with the closed and open states forming clear free-energy minima in all independent runs. The four half-closed states (Figure 4) also constitute local minima, albeit with relatively shallow basins, and the transition pathways between closed and open states clearly proceed through them. Consistent with the standard simulations, the closed state isomer I/open state transitions prefer the half-closed state IL, while IIR is preferred by isomer II/open state transitions. This suggests that the DNA sequence affects the transition pathway preferences.

**Figure 6:**
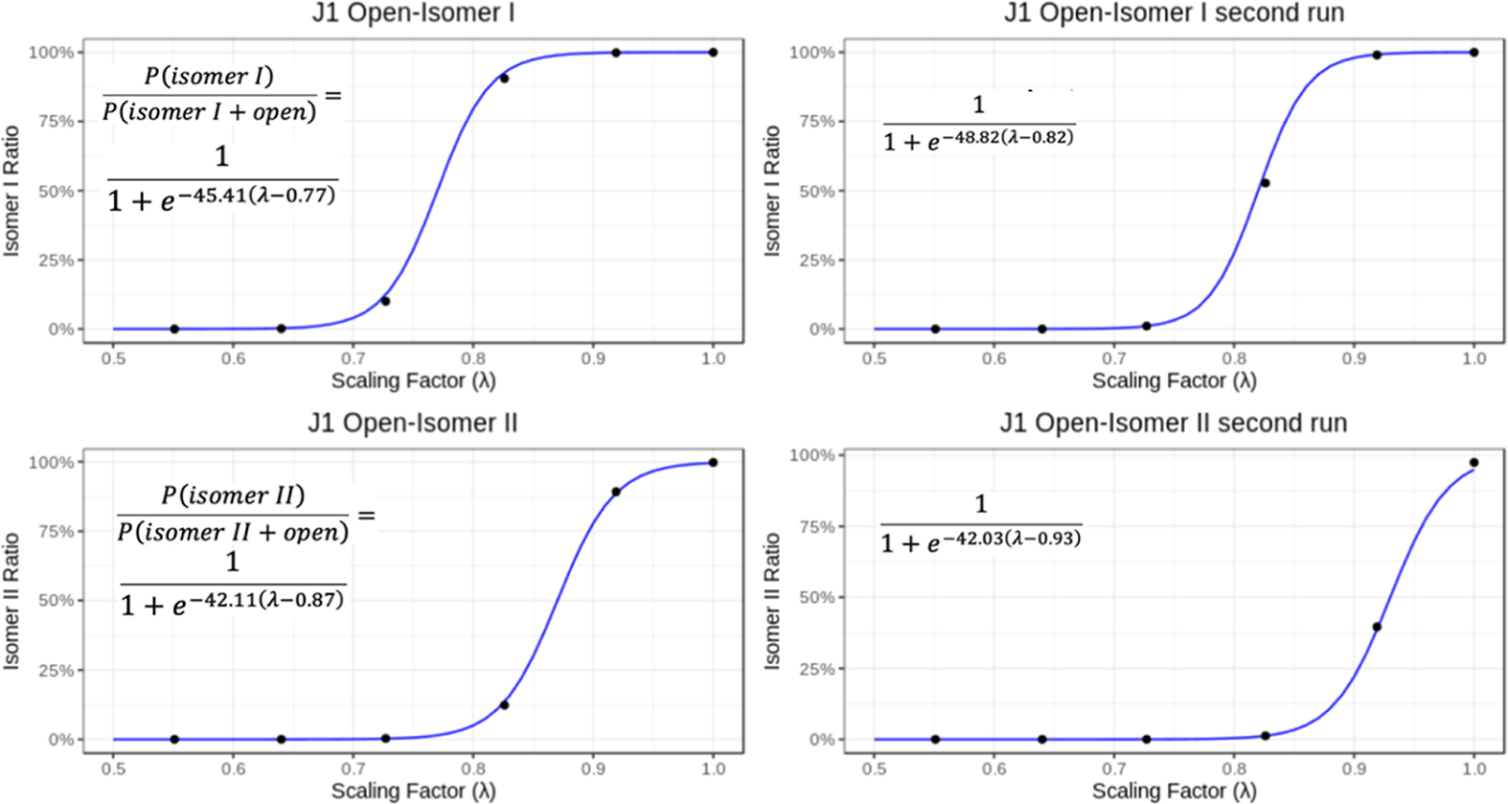
Expected population ratios between the open state and isomers I and II in J1 simulations depending on the scaling factor λ. The values (black dots) were derived from free-energy profiles of the WT-MetaD-HREX runs (Figures 5 and S2) and were used for linear fitting of the Δ*G*_isomer I/II-open_ dependence on λ with the function . This was then turned to the sigmoid ratio expression forms , which are shown as the blue curves. The final expressions of the visualized sigmoid functions are shown in the figure, with the b parameter corresponding to λ, where Δ*G*_isomer I/II-open_ = 0.

The free-energy values of the HJ conformational states in WT-MetaD-HREX are summarized in Table S2. For Isomer I, the two independent simulations predict ideal λ (see Methods) of 0.77 and 0.82, respectively (Figures 5C,D and 6). These values are in good agreement with the value of 0.75 predicted by the standard simulations. Furthermore, the free-energy difference between the closed isomer I and open states with the unmodified force field (λ = 1) obtained by the first and second runs was −6.4 and −5.4 kcal/mol, respectively. This confirms a large overstabilization of the closed state by the original force field, which essentially prevents the occurrence of even a minor population of the open state. The free energies of the half-closed intermediates are also quite high without the scaling, explaining the lack of any opening events (even unsuccessful ones) observed in standard MD simulations without scaling. Note that only the HJ state population ratios differ among the replicas, while the preferred opening–closing pathways and the positions of the basins remain the same, suggesting that the transition mechanism is not qualitatively affected by our scaling protocol. The difference between the two independent runs reflects genuine convergence uncertainties of contemporary enhanced sampling methods, as discussed elsewhere.^61^

The WT-MetaD-HREX simulations of the transitions between the isomer II closed state and open state provide ideal λ of 0.87 with a free-energy difference under unmodified Hamiltonian −3.6 kcal/mol for the first run and λ = 0.93 and −2.2 kcal/mol for the second run (Figures 5E,F and 6). The simulations thus indicate (Figure 5, Table S2) that isomer I is more stable than isomer II by ∼3 kcal/mol on average. This trend is consistent with the experimental preferences reported earlier^16,25,26^ as well as the sampling frequencies of the two isomers we observed in standard MD (Figure 4). We need to caution, however, that because we could not make a four-dimensional MetaD simulation which would directly bridge both isomers, it is not guaranteed that the open state is sampled equivalently in the two WT-MetaD-HREX setups, although it is likely to be so. Therefore, the estimate of the isomer I vs isomer II relative stability carries a small uncertainty. We note that the system is complex, and it is thus very difficult to pinpoint potential differences between the two open state ensembles. Any dimensionality reduction or clustering procedure might further hide those differences. The only rigorous way to claim equivalence of the two ensembles would be to sample continuous trajectories connecting the two isomers, which is not possible with the utilized methodology.

In summary, the WT-MetaD-HREX results fully explain the lack of opening with the standard force field, as well as the intricate conformational interrelations among the open state, the two closed states isomers, and the half-closed states (Figure 4). Note that the shallow local free-energy minima for the half-closed states and their short lifetimes in the standard simulations suggest that these intermediates are rather transient. Given the resolution limits of experimental methods, this might be the reason why the half-closed states have so far not been reported in the literature. We hypothesize the half-closed states could potentially be targeted by some proteins interacting with HJs. However, we are currently aware of no such structure in the database. The above computations were done under net-neutralizing conditions with 230 mM concentration of K^+^ in the box. We have subsequently used WT-MetaD-HREX to investigate the HJ dynamics in response to different ion concentrations. These computations confirm the sensitivity of the free-energy balance between open and closed states to the cation concentration, while also highlighting the limitations of comparing the effective simulation bulk ion concentrations with experimental values (see below).

### WT-MetaD-HREXs of HJ J1 in Different Cation Concentrations

The balance between open and closed HJ states is experimentally known to depend on cation concentration. Namely, the open and closed states tend to dominate at low and high salt conditions, respectively (see above). Therefore, we wished to verify whether this trend is still maintained in our HJ simulations even after applying the scaling. To that end, we performed three additional WT-MetaD-HREX calculations of the J1 isomer I/open state transitions with *c*(K^+^) equal to 1 M (high salt) and 150 or 90 mM (low salt), respectively. Note that the *c*(K^+^) = 150 mM and *c*(K^+^) = 90 mM simulations were performed at net-neutral conditions with the lower K^+^ concentrations obtained by greatly expanding the size of the simulation boxes (see Methods). For the *c*(K^+^) = 1 M, excess-salt KCl was added while using standard box size.

**Figure 7:**
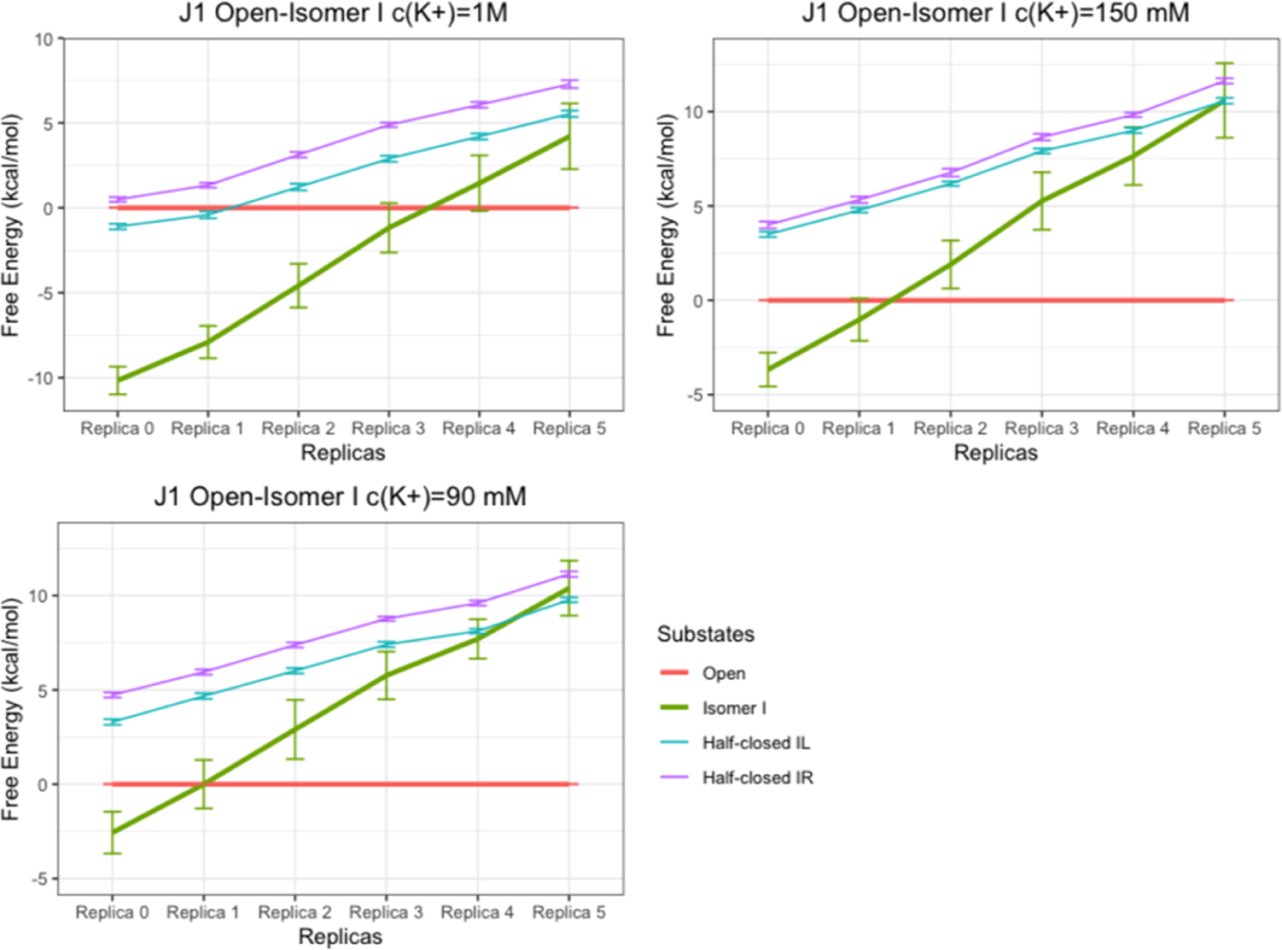
Free-energy difference of each HJ substate to the open state in the replica ladder (with different λs applied for the branch point nucleotides) in the WT-MetaD-HREX systems with *c*(K^+^) = 1 M and 150 or 90 mM (see above for details). The free-energy values of each state were calculated by integrating the probability density in the corresponding CV region with error bars calculated by bootstrapping analysis. We used the open state as reference in each replica, and thus, no error bar is shown for the red line. See also Table S2.

At all three ion concentrations, the free-energy landscapes show clear minima for the same HJ substates as the previous WT-MetaD-HREX (*c*(K^+^) = 230 mM, net neutral) calculations discussed extensively above. This confirms that varying the cation concentration does not alter the opening–closing mechanism in a qualitative way. On the other hand, the ratio between the open and closed HJ state populations is different (Table S2). Namely, in the system with *c*(K^+^) = 1 M, the junction has deeper free-energy basin for the closed isomer I in the lower replicas. In contrast, the HJ under lower cation concentrations (150 and 90 mM) increasingly populates the open state with the corresponding Δ*G*_isomer I-open_ increased in all replicas (Figure 7). This confirms that the experimentally known influence of cation concentration on the populations of HJ states is fully maintained by our scaling model.

**Figure 8:**
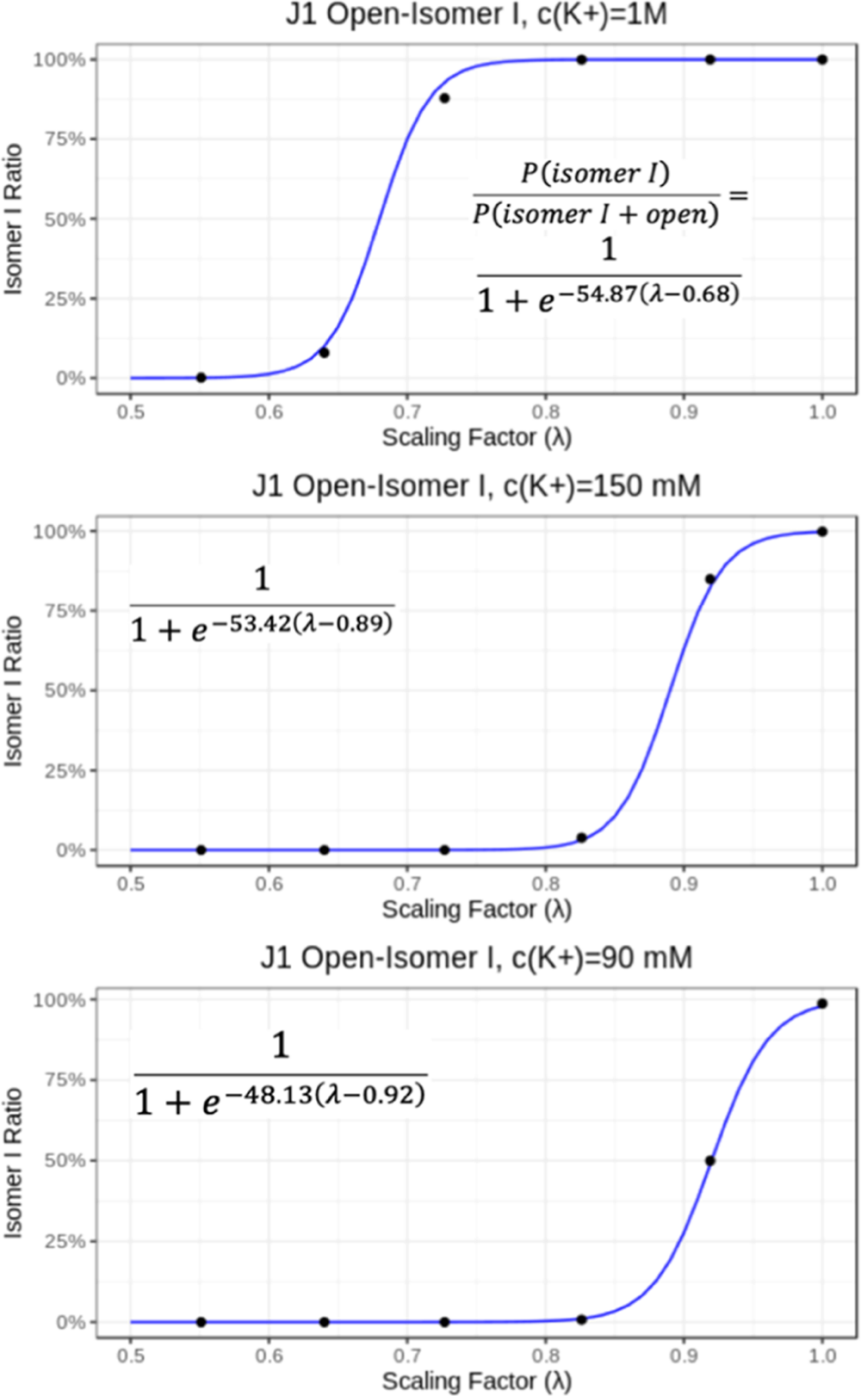
Expected population ratios between the open state and isomers I in J1 simulations under different *c*(K^+^) depending on the scaling factor λ. The values (black dots) were derived from free-energy profiles of the WT-MetaD-HREX runs (Figure 7), which were used for linear fitting of the Δ*G*_isomer I-open_ dependence on λ, using the function . This was then turned to the sigmoid ratio expression form with equation . The final sigmoid functions are visualized in the figure; *b* variable corresponds to λ, where Δ*G*_isomer I-open_ = 0.

Importantly, the additional WT-MetaD-HREX calculations confirmed the overstabilization of the closed state by the force field as the unscaled replica (replica 0) shows the closed state being clearly preferred even at *c*(K^+^) = 90 mM (see also the next paragraph). This demonstrates the usefulness and necessity of our scaling protocol. Furthermore, it should be noted that the ideal λ, at which the Δ*G*_isomer I-open_ = 0, shifts to higher and lower values at low and high cation concentrations, respectively (Figure 8). Therefore, the calculations suggest that the ideal scaling factor would have to be adjusted depending on the utilized cation concentration in order to achieve equal stability of the open and closed HJ states and to see the transitions (Figure S12). This is the expected result and confirms that the simulations properly respond to the changes of the cation concentration.

### Bulk Concentration of Cations Is Underestimated with Net-Neutralizing Simulation Conditions

The cation concentration in explicit-solvent MD is standardly calculated as the relationship between the number of cations present and either the volume of the simulation box or the number of water molecules. The latter approach was used in this work. However, it must be noted that neither of these approaches expresses the bulk ion concentration as measured in experimental settings.^31,52^ This is because the small box sizes and the periodic boundary conditions utilized in MD simulations result in high solute concentration and lack of bulk solvent in simulations. Since our HJ systems are net-neutralized with K^+^ (i.e., there are no anions), the ion concentration calculated by the above-mentioned methods actually refers to the ionic atmosphere of the DNA rather than the bulk.^53^ This obscures the comparison with the experiments where one cannot separate this ionic atmosphere from the DNA in solution without an external electric field applied. In fact, the net-neutral simulation conditions can only be directly related to a hypothetical experiment with zero bulk concentration of ions.

**Figure 9:**
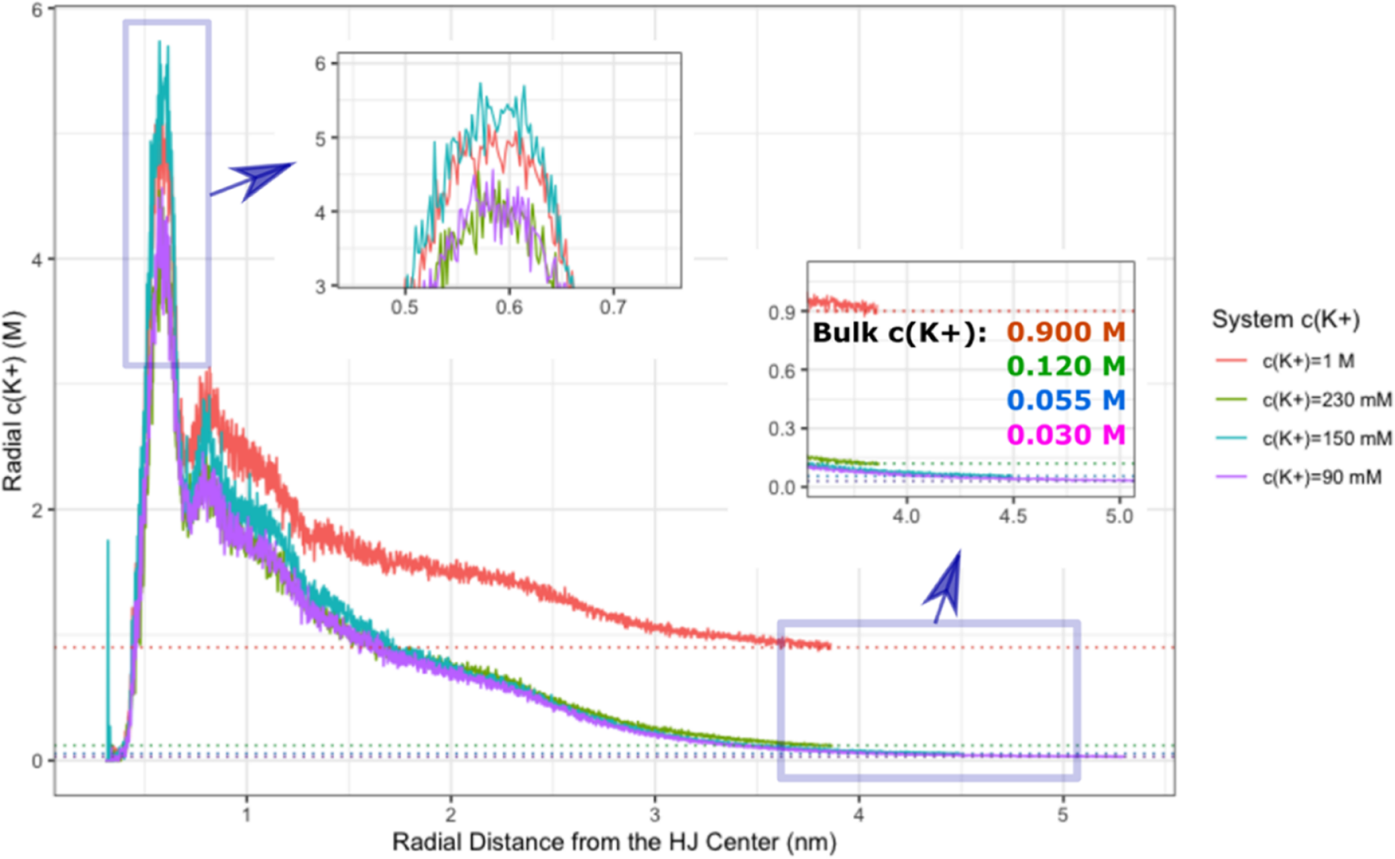
Effective bulk concentrations of potassium in WT-MetaD-HREX simulations. The radial *c*(K^+^) at specified distance from the phosphorus atom joining the arms I and IV of the HJ (referred to as “HJ center”) is estimated from the RDFs of water oxygens and K^+^. The bulk concentration values of the systems with different *c*(K^+^) are shown as horizontal dotted lines and describe the average radial *c*(K^+^) near the edge of the simulation box. The bulk *c*(K^+^)s calculated in this fashion greatly differ from the values obtained by standard calculations involving the number of K^+^ and the water molecules.

To give some estimations of effective bulk (or bulk-like) ion concentration in MD simulations, we focused on a region near the edge of the simulation box with the assumption that the concentration of ions in this region approximates the bulk concentration as close as possible (Figure 9). We observed that all systems had these bulk concentrations well below the values obtained by the standard calculation mentioned above. Namely, for *c*(K^+^) values 1 M, 230, 150, and 90 mM (calculated in relation to the number of water molecules), the effective bulk concentrations are lowered to 900, 120, 55, and 30 mM, respectively. This is because the negatively charged DNA attracts most of the net-neutralizing potassium. We also noticed that the effective bulk concentrations can vary for the closed and open HJ conformations as the closed conformation tends to attract a greater number of cations toward the HJ center. Namely, in the simulations with *c*(K^+^) = 1 M, 230, 150, and 90 mM, the bulk concentrations are 900, 110, 54, and 30 mM for the closed HJ, respectively, while they increase to 1 M, 150, 75, and 50 mM for the open HJ.

The reason we resorted to net-neutralization in our simulations is that including a reasonable amount of anions, e.g., Cl^–^, would have required extremely large simulation boxes and slowed down the simulations beyond the sustainable level. In fact, to make the DNA simulations fully correspond to a typical experimental bulk environment, one would have to increase the simulation box size, so the anions (e.g., Cl^–^) can be included and *c*(K^+^) ≈ *c*(Cl^–^), all with simulation boxes large enough that the experimentally relevant ion concentrations are properly reproduced. Such a large box would necessitate running very short simulations and thus result in poor statistical significance of the results. However, despite these limitations, we still show that the trend in ion concentration effects observed in our WT-MetaD-HREX calculations is in agreement with the experiments (Figures 7 and 8). Considering that the true effective ion concentration can be expected to be between the common box *c*(K^+^) and bulk-like values calculated above, the overstabilization of the closed form by the force field is actually more pronounced than the standard ion concentration calculations would suggest. That is why we can expect that the standard net-neutralizing K^+^ box (230 mM standard calculation/120 mM “bulk” estimate) should already be providing similar stabilities for the open and closed HJ forms and transitions should be observed. Lastly, given that our WT-MetaD-HREX computations provided us with Δ*G*_closed-open_ as a linear function of λ, another arbitrary free-energy value can be chosen as a target to derive the corresponding λ.

### Standard MD of J2 and J13 Junctions Reveal Sequence-Dependent Effects on the Opening–Closing Dynamics

**Figure 10:**
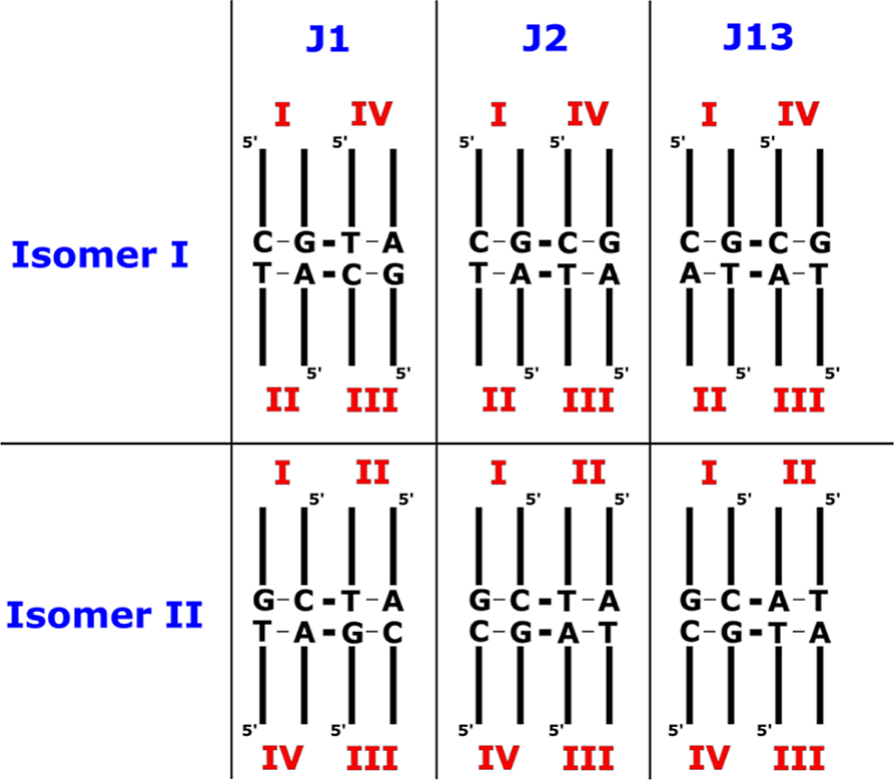
Scheme of the J1, J2, and J13 junctions. A different identity of the branch point base pairs in J1, J2, and J13 junctions is indicated. The rest of the DNA sequence is identical in all three junctions.

The above results describe the J1 junction, the most studied immobile HJ. However, overstabilization of the closed state by the standard force field is likely to affect all HJs. At the same time, the ideal scaling factor λ which allowed us to achieve a more balanced dynamics description of J1 is highly empirical and could vary for other HJs with different identities of the branch point nucleotides and thus different stacking partners. We therefore performed additional simulations of the J2 and J13 immobile HJs (Figure 10).^14,26^ We found out that the ideal λs under which both closing and opening transitions can be observed are indeed variable, with λ being 0.75, 0.775, and 0.8 for J1, J2, and J13, respectively. Although the differences are seemingly not large, they are significant since 0.025 difference in λ is equivalent to ∼1.12 kcal/mol vdW interaction energy difference for the closed junction (Figure S1). In fact, we have seen that the ideal λ value of 0.775 for J2 already inhibits the opening dynamics for J1.

The preference for the closed state isomer I or II may also vary among the junctions, as indicated by the experiments.^26^ Indeed, in our J2 simulations, we observed 6 closing events, 4 of them to isomer I and 2 to isomer II. J13 simulations showed 10 closing transitions to isomer I and only 2 to isomer II. In J1 simulations, only 4 closing events out of 26 led to isomer II (Figure 4), which are consistent with the WT-MetaD-HREX results, suggesting deeper free-energy basin for isomer I. The standard MD results are obviously not quantitatively converged and primarily reflect the probability of the closing events rather than the equilibrium between isomers.

### Parallel Conformations of HJ

In some of the standard MD simulations, we observed that the closed HJ could temporarily flip the arrangement of its helical arms from the native antiparallel arrangement into a parallel one. Although structurally feasible in principle, experimental studies showed that the parallel HJ is not nearly as populated in solution as the antiparallel one;^16,28^ however, it can be reached during fluctuations.^62^ In all of our simulations, we observed the parallel conformation of HJ in ∼10% of time, which could possibly be excessive and reflect additional inaccuracies in the force field (Table S3). We stress that the transitions between the antiparallel and parallel forms are fully reversible. However, in some cases, we observed extensive interhelical interactions in the parallel forms, which slow down the transition back into the antiparallel form (Figure S3). This was much more common in our simulations of HJ constructs with the truncated helical arms, but quite rare in the full-length HJ systems, such as those that we utilized for our enhanced sampling calculations. For these reasons, we strongly recommend that HJs constructed with longer helical arms (at least eight base pairs per arm) are preferred for future simulation studies. We also note that for the purposes of our study, the parallel HJ conformation is not of interest because the opening–closing transitions occur only with the antiparallel structure. We plan to explore this more in future studies.

### Comments on the Computational Methods Used

We have achieved spontaneous opening–closing dynamics of HJ by developing a system-specific correction for the AMBER force field, which radically weakens the vdW interaction of the branch point nucleotides. The specific λ value is derived together with the OPC water model and the CUfix parameters, which weaken the ion–pair interactions.^37,63^ Our modification is not a general reparameterization of the force field. However, the approach could be used to introduce suitable weakening of vdW interactions (mainly stacking) for some other DNA systems and simulation conditions, when necessary. Note, however, that extensive simulation tests to derive the ideal λ value would be required.

Our approach can be justified in the following way. Considering the magnitude of the necessary force-field correction to simulate the target process, attempting to reparameterize the general force field could be an unrealistic goal. In addition, the imbalance in description of the vdW interaction is context- and system-dependent, as evidenced by the ideal λ values being different for the three HJ sequences we tested. Furthermore, we suggest that the current simple force-field form is close to the limits of its capability to be further improved.^31^ In other words, we suggest that a goal to develop a simple all-atom AMBER-type force field that would be universally correct for all nucleic acids forms and in all situations is not realistic. Thus, instead of the daunting search for the universal force field, we decided to develop a goal-specific modification. Although such an approach may look at first sight unsatisfactory, the same strategy is standardly used in the context of coarse-grained modeling and can be fully justified also in the framework of the empirical all-atom description methods.^64^ As a further control, we performed the simulations of B-DNA using scaled vdW interactions *via* the same scaling protocol utilized for HJ. Results are reported in Supporting Information and show that the protocol does not lead to any perturbation of B-DNA simulations on the μs timescale.

Furthermore, there could also be some concerns that the scaling protocol may introduce some biases; for instance, the possibility that the half-closed intermediates arise as a consequence of the introduced scaling modification. Nevertheless, the half-closed intermediates are seen also in the pure WT-MetaD simulations carried out without any scaling. Although not converged, this supports the existence of half-closed intermediates even in the absence of any scaling (see Supporting Information for details). We also observed half-closed intermediates during the HJ closing in unscaled standard MD simulations started from open HJ structure. Note that the HJ opening transitions cannot be observed in standard MD without the scaling. Obviously, we do not suggest that our force-field variant provides a perfect description of the HJ dynamics. One possible caveat could be the rather fast kinetics of the opening–closing dynamics (microseconds in MD vs the milliseconds observed experimentally).^16,17,24^ The simulations should nevertheless be sufficient to provide a qualitatively correct atomistic picture of the opening–closing transitions, something which was originally not possible.

The work is based on a combination of standard and enhanced-sampling simulations. It is encouraging to see that the two approaches complement each other and provide a similar picture of the HJ dynamics. Nevertheless, the sophisticated WT-MetaD-HREX approach seems to suggest a slightly lower degree of scaling (λ closer to one), which can be caused by several factors. In the limit of very large sampling, standard simulations would be considered as the benchmark for the enhanced sampling simulations. However, the present calculations are far from converged sampling, and thus, the enhanced sampling approach is expected to broaden the sampling. On the other hand, some enhanced sampling methods can introduce bias, especially when reducing dimentionality with CVs.^43^ We also do not sample the junction branch migration attempts in our simulations, which can compete with isomerization since both processes have the open state as an intermediate. Although modern enhanced sampling methods offer a decent way to improve sampling, they are not a panacea. For example, a recent comprehensive study of the folding landscape of simple RNA tetraloops using a similarly sophisticated combination of RE method with MetaDynamics suggested that the true uncertainty in the estimated folding free energies due to convergence could be around 1 kcal/mol.^61^ This is consistent with the difference between two independent WT-MetaD-HREX runs found in the present study. We suggest that the present HJ simulations are done at the limits of what one may achieve with contemporary simulation methods and hardware.

## Discussion

In this work, we explore the pathways by which immobile HJs transition between the open and closed conformational states. These transitions were for a long time known to occur in HJs where they facilitate the exchange between closed state isomers I and II as well as the branch migration, both of which are essential in biological processes such as the DNA repair and meiosis.^3,5,16,35,65^ At the same time, the atomistic picture of the transitions was unclear due to their fast timescales.^16,17^ Commonly assumed to be a simple two-state process, we used standard MD and enhanced sampling simulations to show that the opening–closing transitions actually involve previously unconsidered “half-closed” state intermediates (Figure 4) through which the vast majority of the transitions proceed, challenging the commonly assumed simple two-state model. The combined simulation methods we used in this study could function as promising tools to promote the HJ structure and dynamic exploration with new angles of view.

The current state-of-the-art DNA force fields have issues in simulating HJ opening–closing dynamics as the closed state is overstabilized even under the low cation concentration environment.^33,35^ The overestimated cation–phosphate interaction and vdW interactions of the branch point nucleotides are the most likely hurdles of the open state HJ simulations.^37,39,40,66^ Here, we showed that both of these issues have to be addressed since merely weakening the cation contacts with the junction branch point phosphate groups^37,50,51^ is insufficient to achieve HJ opening. By scaling down the LJ potential well depths among the branch point nucleotides, we for the first time realized the spontaneous opening and closing transitions sampling in standard MD simulations, paving a way to future studies of the HJ conformation transitions and even the branch migration processes. Moreover, the different ideal scaling factors (λs) determined for J1, J2, and J13 confirmed the sequence effects on the junction opening–closing dynamics. The observed transitions suggested two potential routes between each closed state isomer and open state *via* the corresponding half-closed states. We also observed the system preference for one of the two transition pathways and the stability difference between the two closed state isomers of HJ in standard MD. These are further supported by the enhanced sampling simulations which explained the phenomena detected in standard MD simulations, confirmed the necessity of applying the λs on the branch point nucleotides, and detailed the sensitivity of HJ dynamics to the cation concentrations. We note that this procedure does not introduce any obvious deviations from canonical B-DNA structure (Supporting Information). However, the scaling is still system-specific, as shown by the comparison among J1, J2, and J13. It could be unrealistic to develop perfect force-field parameters correctly describing LJ interactions in all DNA structures and would be especially complicated for HJs due to complexity of their interactions, including the electrostatic interactions among the branch point base pairs, the topology restraints given by the crossover strands, and the ion binding. All of these critically influence the HJ dynamics.^17,18,22,34,37,40^ The scaling strategy we used in this study has perspective for more extensive *in silico* studies on HJ dynamics, not limited to the 36 immobile HJs anymore, but moving on to the mobile junctions which are capable of branch migration. The migrating HJs are difficult to study but more biologically relevant than the immobile sequences. Detailed knowledge of the junction transition pathways could assist our understandings of essential biological processes such as the HJ recognition by enzymes which might involve specific HJ conformation as the target.^3,5,16,35,36,65^ We, for example, expect that some proteins could be specifically recognizing the half-closed states as part of HJ resolving or protein-assisted branch migration. The opening–closing dynamics of HJ and the isomerization preference unveiled by the computational methods could also benefit the DNA nanotechnology where HJ is a common building stone whose dynamic properties have dramatic influence on the successful formation of the 3D lattices.^13,14,25,26^ Using the WT-MetaD-HREX strategies presented in this work to explore the sequence effects of HJs could lead to more reliable designs of HJ-based DNA nanomaterial frameworks with greater stability and variety. Detailed analyses of different base–base stacking identities and even the base pair step parameters could facilitate the sequence programming for HJ-based nanomaterial design.^26^

## Methods

### Standard MD Simulations

We used X-ray structure of the immobile junction J1 in closed state isomer I (PDB: 5KEK)^42^ as the starting structure for the simulations of the closed state. The length of the helical arms was selected to be either 6 or 8 base pairs (Figure 2), with the corresponding number of DNA nucleotides excised from the 3D lattice of the experimental structure.^42^ After the first closed-to-open conformational transition in MD, we extracted several different simulation snapshots of the open conformation and used them as initial structures for the simulations of the open state. Conformations with alternative orientations of the helical arms and a structure near to the intermediate state (“half-closed” conformation) were also used as starting structures of selected simulations. For a full overview of all the utilized starting structures of the J1 junction, see Figure S4. Starting structures of the J2 and J13 junctions were obtained by modifying the branch point base pairs in the J1 structure.

All simulations were conducted with the AMBER18 package.^67^ We used OL15 force field (one of the recommended AMBER force fields for DNA)^45−47^ to describe the HJ along with modified phosphate parameters from the study of Steinbrecher et al.^49^ The OL15 force field was recently shown to perform well in simulations of all 36 immobile HJ sequences in the closed state.^14^ The DNA was solvated in a truncated octahedral OPC water box^63^ with the shortest distance to the boundary being at least 12 Å. For the initial simulations, we established a net-neutralizing K^+^ environment, which, given the size of the simulation box, corresponded to *c*(K^+^) = 240 mM. See also the discussion of differences between experimental and simulation ion concentrations in Results. We have initially used either the Joung and Cheatham (JC)^50^ or Li and Merz (LM)^51,68^ ion parameters, while most of the subsequent simulations were performed with the LM parameters. We also tested 0.15, 0.05, and 0.02 M cation concentrations; when the number of explicit cations was insufficient to fully neutralize the system, the net-neutrality was enforced with uniform neutralizing plasma. In almost all simulations, we applied CUfix modification to increase the optimal vdW distance between K^+^ ions and phosphates.^37^ The complete list of ion conditions is shown in Table S1. Structure-specific HBfix potential of 2 kcal/mol was applied to the terminal base pair H-bonds in all four helical arms to prevent their fraying.^69^ This included the branch point base pairs which become helix-terminal during the junction opening. Although terminal base pair fraying, especially of the A–T base pairs, is a real phenomenon, it is likely exaggerated by the force field.^31,70^ The fraying would also significantly reduce the sampling effectiveness of the immobile HJ simulations (Figure S5, see Supporting Information for more details). Prior to the production simulations, all systems underwent minimization and equilibration steps (Supporting Information). The standard simulation length for each production simulation was 1 μs, while some simulations were extended up to 10 μs.

### Scaling of the LJ Potential among the Branch Point Nucleotides of the HJ

In order to attenuate the vdW interactions at the HJ center, and thus to allow the opening–closing dynamics, we adjusted well depths (ε) of selected pairwise LJ interactions (see Supporting Information for more details) among atoms of the central nucleotides, including sugars and phosphate groups, by multiplying them with a uniform scaling factor (λ); we tested λ values ranging from 0.5 to 0.9. The H-bond donors and acceptors involved in the Watson–Crick base pairing interactions were not scaled in order not to affect the stability of the base pairs. Our scaling protocol is very similar to the one recently proposed to eliminate excessive intramolecular interactions in RNA simulations.^71^ The approach corresponds to the NBfix method but is applied in a structure-specific manner and only in the region of interest.

The ideal λ was determined empirically using a series of standard simulations with different λs, searching for a value that leads to spontaneous opening and closing dynamics in standard simulations.

After determining the approximate range of λ which allows both closed-to-open and open-to-closed transitions, we performed additional simulations to refine the λ. Overview of all simulations with different λs is in Table S1. The ideal λ found in this way should be close to the one leading to zero free-energy difference between the open and closed states (see also the next paragraph) though we cannot confidently estimate free energies from the standard simulations.

### Well-Tempered MetaDynamics with Hamiltonian Replica Exchange

The enhanced sampling simulations were performed using a method combining Well-tempered MetaDynamics with Hamiltonian Replica Exchange, abbreviated as WT-MetaD-HREX. The same system building protocol and equilibration as in standard MD was applied. The AMBER input files were subsequently converted to GROMACS input files by ParmEd, and the WT-MetaD-HREX was performed with GROMACS-v2018^72^ and Plumed-v2.5.6.^73^

The WT-MetaD-HREX was done in a two-dimensional CV space. Coordination numbers between the branch point base pairs of the HJ (Figure 2) were used as the CVs to boost sampling of the opening–closing transitions. These CVs unambiguously distinguish closed, open, and half-closed HJ states by quantifying the short-range contacts between two groups of atoms, in our case the base stacking. The nucleobase atoms of the four branch point base pairs were assigned into four groups. We calculated the coordination numbers of the group combinations, labeled as sI-II, sIII-IV, sI-IV, and sII-III (stem numbers defined in Figure 2), describing all four base pair stacks that can potentially occur at the HJ center. The applied switching function is a rational function (eq 1) with *n* = 4 and *r*_0_ = 3 Å parameters (Figure S6). We considered the HJ to be in the open state when all four CVs are below 5, while the closed state isomer I or II was present when their respective CVs are over 50. Finally, the half-closed states were considered present when one of the four CVs is over 50 and the three remaining ones below 5.

We performed 2D WT-MetaD-HREX calculations with two different CV combinations. The CVs sI-II and sIII-IV were used for the sampling between open state and isomer I. The isomer II-open state sampling was described by sII-III and sI-IV CVs (Table S4). The two groups of CVs were normalized by exponents 0.65 and 0.4 for isomer I-open and isomer II-open simulations, respectively, in order to have similar standard deviations of CVs in open and closed states (Table S5) and to achieve a good MetaD convergence. The ranges of CVs explored in WT-MetaD-HREX were 0/0 to 26/26 and 0/0 to 8/8 for sI-II^0.65^/sIII-IV^0.65^ and sI-IV^0.4^/sII-III^0.4^, respectively. In order to avoid sampling of isomer II in isomer I-open simulation or *vice versa*, two parabolic energy restraints on the CVs leading to the other isomer were set for corresponding coordination numbers starting from 10 and above, with a force constant of 0.02 kJ/mol. Weighted Gaussians in the CVs’ profile were added every 500 steps (every 2 ps), with the initial height of 2.5 kJ/mol, the σ values of 0.8/0.8 and 0.2/0.2 determined by the standard deviation of the CVs (Table S5) sI-II^0.65^/sIII-IV^0.65^ and sI-IV^0.4^/sII-III^0.4^, respectively, and the bias factor equal to 15. The potentials added to the CV space were averaged and reweighted by PLUMED to compute the free-energy profile in the 2D-CV space (see Supporting Information). We point out that, due to computational cost, only two CVs can be included in each WT-MetaD-HREX, so that we decided not to use single set of WT-MetaD-HREX to cover the entire isomer I-open-isomer II HJ dynamic space. The sampling was further boosted by the HREX, where we set a ladder of scaling factors (λs) across 6 replicas for the LJ potentials among the branch point nucleotides (see above and Table S6). In other words, the HREX ladder is using exactly the same modification of the Hamiltonian which we designed to overcome the overstabilization of the closed state in standard simulations of the HJ. This also allows us to estimate (interpolate) the ideal values of λ from the HREX ladder corresponding to zero free-energy difference between the closed and open states. The exchanges along the ladder were attempted every 2500 steps (10 ps) with the exchange acceptance determined by eq 2, where *U*_*i*_ is the potential energy operator in replica *i* and *x*_*i*_ is the corresponding coordinate.

The simulations started from either 64 nt isomer I or II, and each ran for 1 μs. The initial structure of isomer II was obtained by steered MD simulation started from the open structure, see Supporting Information for details. Two independent simulations were executed for each WT-MetaD-HREX setup in order to estimate the uncertainty of WT-MetaD-HREX computation. These WT-MetaD-HREX runs were done with 230 mM concentration of net-neutralizing K^+^.

Three additional WT-MetaD-HREX systems with *c*(K^+^) = 1 M and 150 and 90 mM, respectively, were also built for the J1 isomer I to investigate the scaling effects under different cation concentrations. The net-neutral 150 and 90 mM systems were prepared by building a larger water box in leap, with minimal distances between the solute and the box border of 21 and 29 Å, respectively. The 1 M simulations were performed with the standard box size under excess-salt conditions (i.e., including the Cl^–^ anions). The list of all WT-MetaD-HREX simulations (Table S4), details of the free-energy analysis, and some other information are provided in the Supporting Information. We initially also tried applying a pure HREX method to simulate J1. However, without adding the CV-based method, the simulations did not show any sign of convergence; for more details, see the Supporting Information.
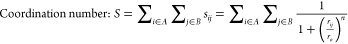
1
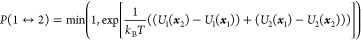
2

### Analyses

The MD trajectories were visualized, and conformational transitions of HJ were evaluated with VMD (version 1.9.3), PyMOL (Version 2.0 Schrödinger, LLC), and UCSF Chimera. The details of the systems were extracted by PLUMED-v2.5.6^73^. and cpptraj,^75^ processed in RStudio and visualized by ggplot2.

## Data Availability

All the data necessary to support and reproduce the findings of this study are available in this text and in the Supporting Information. All the data including the simulation input and parameter files, the bias files from the WT-MetaD-HREX, and the output files for generating the results have been published in Zenodo repository (10.5281/zenodo.7684315). The PDB files of simulation starting structures (Figure S4) are attached in the Supporting Information. The AMBER18 package and OL15 force field can be licensed and downloaded from AMBER (http://ambermd.org/) and OL Force Fields official webpage (https://fch.upol.cz/ff_ol/downloads.php). The GROMACS-v2018 is available for free (https://www.gromacs.org/). PLUMED-v2.5.6 is available for free (https://www.plumed.org/download/), and the WT-MetaD-HREX protocol and analyses PLUMED files are freely downloaded from either GitHub or PLUMED Consortium,^76^ Plumed-Nest (https://github.com/sponerlab/WT-MetaD-HREX_HJ or https://www.plumed-nest.org/eggs/22/036/). The PyMOL Molecular Graphics System can be licensed from Schrodinger (https://pymol.org/2/). The VMD molecular visualization program can be licensed from UIUC (http://www.ks.uiuc.edu/Research/vmd/).

## Supporting Information Available

Initial HJ structures used for the MD simulations (ZIP)
More detailed simulation setups, additional enhanced sampling protocol, and HJ simulation data and analyses (PDF)
