# Supplementary material for: Atomistic Picture of Opening–Closing Dynamics of DNA Holliday Junction Obtained by Molecular Simulations
Source: J Chem Inf Model. 2023 Apr 26;63(9):2794–809. doi: 10.1021/acs.jcim.3c00358 (PMC10170514; doi:10.1021/acs.jcim.3c00358)
Supplement: Supplementary file 2 — ci3c00358_si_002.pdf [file ci3c00358_si_002.pdf]

Supporting Information for:

Atomistic Picture of Opening-Closing Dynamics of DNA Holliday Junction Obtained by Molecular Simulations

Zhengyue Zhang[1,2,3], Jiří Šponer[1], Giovanni Bussi[4], Vojtěch Mlýnský[1], Petr Šulc[5], Chad R. Simmons,[5] Nicholas Stephanopoulos[5], and Miroslav Krepl[1,6]*

[1] Institute of Biophysics of the Czech Academy of Sciences, Královopolská 135, 612 00 Brno, Czech Republic

[2] CEITEC − Central European Institute of Technology, Masaryk University, Kamenice 5, 625 00 Brno, Czech Republic

[3] National Center for Biomolecular Research, Faculty of Science, Masaryk University, Kamenice 5, 625 00 Brno, Czech Republic

[4] Scuola Internazionale Superiore di Studi Avanzati (SISSA), via Bonomea 265, Trieste 34136, Italy

[5] Biodesign Center for Molecular Design and Biomimetics, 1001 S. McAllister Ave. Tempe, AZ 85287, Arizona State University, USA

[6] Regional Centre of Advanced Technologies and Materials, Czech Advanced Technology and Research Institute (CATRIN), Palacky University Olomouc, Slechtitelu 241/27, 783 71 Olomouc, Czech Republic

Corresponding author: Miroslav Krepl, krepl@ibp.cz.

Supporting Text

Details of the molecular dynamics (MD) simulation preparations

The standard and enhanced sampling MD simulations were carried out with AMBER18[1] and GROMACS-v2018[2], respectively. Each system was prepared in Leap module of AMBER with different ion models[3,4] and potassium concentrations (Table S1). CUfix[5] and the phosphate parameters from Steinbrecher et.al[6] were applied in ParmED. Hydrogen mass repartitioning was applied to allow a longer integration time step (0.004 ps) in simulations.[7] Afterwards, each system went through a series of minimizations and equilibrations. The initial minimization of the system was done while applying a 25 kcal/mol/Å^2 positional restraint on the solute atoms. The systems were then heated up from 100 K to 300 K while using the same positional restraint. Afterwards, alternating cycles of minimization and equilibration were implemented for five rounds. In each round, the positional restraint placed on the solute changed from 5 kcal/mol/Å^2 to 1 kcal/mol/Å^2 in decrements of 1 kcal/mol/Å^2. The positional restraint for the final equilibration was 0.5 kcal/mol/Å^2. For each minimization, we ran 500 steps of steepest descent followed by 500 steps of conjugated gradient

minimization. The equilibrations were run for 50 picoseconds each.

The use of HBfix in HJ simulations

HBfix is a structure-specific potential which can be used to adjust stability of selected H-bonds. In case of HJ, we used it to prevent the fraying of the terminal base pairs which is a common phenomenon in simulations of nucleic acids.[8,9] A 2 kcal/mol potential HBfix per H-bond was applied to increase the stability of terminal base pairs in all four HJ arms. This includes the branch point base pairs since they are also termini when the HJ opens and can become frayed at that point. The fraying of branch point base pairs in the open state could represent a genuine attempt for branch migration. Even though we only simulated immobile HJs where the migration cannot succeed, the system can still make such attempts during random thermal fluctuations. Although intriguing, exploration of such states in the context of the present study would have been very challenging in terms of sampling. We plan to explore the mechanism of migration as well as behaviour of migrating HJs in future studies. Note that the HBfix potential in WT-MetaD-HREX simulations was 4 kcal/mol per H-bond instead of 2 kcal/mol as the latter was not enough to keep branch point base pairs from breaking under the effects of MetaD biases, as revealed by the initial calculations (not shown).

Protocol for scaling the LJ interactions of the branch point nucleotides in HJ

The LJ interactions among the branch point nucleotides were modified by scaling the ε parameters (LJ well depth) among the atomic pairs, including the nucleobases, the sugars and the phosphate groups, but excepting the atoms directly involved in the Watson-Crick base pairing H-bonds, i.e. atoms N4, H41, N3, O2 (cytosine), O4, N3, H3 (thymine), N6, H61, N1 (adenine) and O6, N1, H1, N2, H21 (guanine). This approach is similar to the Stafix potential recently proposed by our group which reduces the excessive intramolecular interactions in RNA simulations.[10] The main difference is that Stafix[10] avoids scaling of all the potential H-bond interactions whereas here, we avoid only those involved in the Watson-Crick base pairing H-bonds which occur in the B-DNA duplexes. The ε parameters were scaled by λ ranging from 0.5 to 0.9 until we identified the ideal λ which allows both opening and closing transitions in the standard MD simulation. The ideal λ was determined in two rounds of searching, where the first round was a rough search with the step size of 0.1. Once identifying the approximate λ where transitions in both directions could be observed, we then ran a second round with a more precise search step size of 0.025 to obtain the ideal λ value.

Reference simulations of B-DNA (Dickerson dodecamer) with scaled LJ interactions

We ran additional standard simulations of the Dickerson dodecamer (PDB ID:1BNA, resolution 1.9 Å)[11] to better identify potential side effects of the scaling protocol utilized to reduce the LJ interactions of the branch point nucleotides in HJ (see above). Depending on whether the scaling was used and whether we applied HBfix to stabilize the B-DNA terminal base pairs, four systems of B-DNA simulations were run (Table S1). We used the same preparation and simulation protocol as for the HJ simulations (see above and main text Methods). For all the scaled systems, the scaling factor λ=0.7 was applied for the entire DNA helix. In all simulations, the DNA duplex remained stable, with the helical parameters and base pair stability almost identical in both the scaled and non-scaled simulations (Figure S7 and Figure S8)[12]. In simulations where we did not stabilize the terminal base pairs with HBfix, we observed terminal base pair fraying, regardless of whether the scaling was applied. In other words, the terminal base pair fraying was not significantly promoted by the scaling, even if the scaling was applied throughout the DNA helix. Therefore, we suggest that the scaling protocol we applied for the HJ systems does not deteriorate the B-DNA structure compared to the original force field. Furthermore, the same protocol could be transferable to simulations of some other DNA systems where reduction of the LJ interactions (mainly stacking) would be desirable.

Determination of the scaling factors in replicas of the WT-MetaD-HREX simulations

The same scaling protocol we used to weaken the LJ interactions among the branch point nucleotides in standard MD was also used to modify the Hamiltonian in the individual replicas of our WT-MetaD-HREX simulations (see main text Methods and the section above). We determined the λs ladder by iteratively running 10-ns-long two-replica simulations, attempting 1000 exchanges during the simulation time until we obtained λ with appropriate exchange rate between the neighbouring replicas (accepted exchange rates were between 22% and 28%). This calculation was repeated with each new obtained λ until we derived a replica ladder where the λ ranged from 1 in the lowest replica to 0.55 in the highest replica. In the end, six replicas were suggested by this protocol and their λ values are shown in Table S6. The replica exchange behaviour is also visualized in Figure S9, supporting our chosen replica ladder.

Preparation of the Isomer II starting structure for WT-MetaD-HREX

In the WT-MetaD-HREXs, we used the closed state HJ as the starting structure. However, we did not have available closed state isomer II structure of 64-nt J1 as the standard MD simulations mainly involved the shorter 48-nt J1. Therefore, to obtain it, we used GROMACS+Plumed[2,13,14] to run Steered MD (SMD) simulation[15] starting from 64-nt-open0 structure (Figure S4) in which we forced the junction to close into isomer II. Just as in the WT-MetaD-HREX, the coordination numbers sI-IV and sII-III (without exponential factor) were used as the collective variables in SMD. We forced the sum of sI-IV and sII-III CVs to move from 0 to 180 within 10 ns using a dynamic harmonic restraint with a force constant of 0.04 kJ/mol. The SMD trajectory was visually inspected, and the isomer II structure was selected from one of the frames at the end of the trajectory. The selected structure was then equilibrated by running a standard MD simulation for 5 ns and the final structure of this simulation was then chosen as the starting structure of the WT-MetaD-HREX isomer II-open space sampling.

Free energy analysis of WT-MetaD-HREX

The trajectories obtained in WT-MetaD-HREX simulations were analyzed by reweighting. We obtained the MetaD biases in gaussian hills file for each replica and reweighted them into the non-exponential CV space. The weight of each frame was computed assuming a static bias V(s) computed at the end of the simulation.[16] The static bias was obtained from a time-average of the last 300 ns so as to reduce fluctuations.[17] The bias was then used to compute weights with eq.1, where k_B is Boltzmann constant and T is temperature. The new weights were then used to estimate the probability density as a function of different CV s' (non-exponential CV) with a Kernel-density estimator in eq.2[18], where s denotes the CV and $K(s-s(t),\sigma)$ is the kernel centered at the current value $s(t)$ with a bandwidth σ. In the end, the histogram was converted to the free-energy profile with eq.3. The free energies of discrete states (open/closed/half-closed IL/IR/IIL/IIR) were then calculated using eq.4.

$$w \propto e^{\frac{V(s(t))}{k_B T}} \text{ (eq.1)}$$

$$\langle P(s) \rangle = \frac{\sum_{t=0}^{t_{max}} w(t')K(s-s(t),\sigma)}{\sum_{t=0}^{t_{max}} w(t)} \text{ (eq.2)}$$

$$F(s) = -k_B T ln\langle P(s) \rangle \text{ (eq.3)}$$

$$F(s_{state}) = -k_B T ln\left(\sum_{i \in state} e^{-\frac{F(s_i)}{k_B T}}\right) \text{ (eq.4)}$$

The state free energies were further used to predict the population ratios of the two isomers and the open state in each replica, which was derived from the $\Delta G_{\text{isomer I/II-open}}$ by using eq.5. The population

ratios (P) were defined as $P(\frac{isomer\ I}{isomer\ I +\ open})$ or $P(\frac{isomer\ II}{isomer\ II +\ open})$, and their values in each replica were plotted as black dots into the Figure 6 and Figure 8. The $\Delta G_{\text{isomer I/II-open}}$ dependence on λ was further estimated by linear fitting (eq.6) with negligible error. By combining the results of eq.5 and 6, we obtained the relation between λs and the population of the closed state in form of a sigmoid function, shown in Figure 6 and Figure 8.

$$\frac{P(A)}{P(B)} = e^{-\frac{\Delta G_{A-B}}{k_B T}} \text{ (eq.5)}$$

$$\frac{\Delta G}{k_B T} = a(\lambda - b) \text{ (eq.6)}$$

Errors estimation and convergence of WT-MetaD-HREX

Application of WT-MetaD-HREX flattened the free-energy landscapes of HJ in the CVs spaces with biases so that the junction could freely diffuse between the closed and open states. In order to measure the statistical errors and evaluate the convergence of our WT-MetaD-HREX, we first did the bootstrapping analysis, and then, our WT-MetaD-HREXs were extended for 300 ns without adding new MetaD bias in all the independent runs in isomer I-open and isomer II-open spaces, including the simulations with different ion concentrations (abbreviated as WT-MetaD-HREXs-EXT).

The bootstrapping was done independently in all replicas for all WT-MetaD-HREX setups, estimating the standard deviation of the free-energy difference between each HJ substate and the open state. In each replica, we divided the trajectory uniformly into 20 blocks and rebuilt the trajectory by randomly picking 20 blocks with replacements. Based on the rebuilt trajectory, we calculated the free energies of the substates with the weight of each frame using the protocol described in the previous section. The process of trajectory rebuilding, and free energies calculations was iterated 200 times. In the end, we obtained 200 free-energy samples and derived the standard deviations of free-energy differences for all substates against the open state from the samples. The standard deviations of the free energies (except the open state which was used as the reference) for all 6 replicas in each WT-MetaD-HREX run were plotted as the error bars in Figure 5 C,D,E,F and Figure 7. The statistical errors in WT-MetaD-HREXs are not significant. However, we also note that the true uncertainty in RE simulations is known to be larger than indicated by the statistical errors and needs to be assessed based on entirely independent simulation runs.[19–21]

For WT-MetaD-HREXs-EXT simulations, the previously accumulated biases in all 6 replicas were included as a static bias potential and no new biases were added. The HREX protocol was kept the same. We first verified that the accumulated biases would enable the closing-opening transitions in the WT-MetaD-HREXs-EXT by tracking the coordination number in each continuous trajectory (Figure S10 and Figure S11), which indicates convergence of the previous WT-MetaD-HREX runs. Similar result was observed for the WT-MetaD-HREXs-EXTs in high-salt and low-salt conditions. The HJ transitions were detectable although not in all trajectories. Especially in the first WT-MetaD-HREXs-EXT run of isomer II-open system, HJ in trajectory 0 remained open throughout the simulation. The same was also observed in the trajectory 4 of the second run. The other trajectories showed transitions, but their frequency was rather low compared to the previous WT-MetaD-HREXs. We explain this by the complicated and large-scale conformational change required for the transitions, leading to a slow diffusion in the coordination number space. Meanwhile, the filled free-energy landscapes are also not exactly flat, which is the result of statistical and systematic errors in WT-MetaD-HREX and the complexity of HJ dynamics. Regarding the latter, it should be noted that HJ has more CVs which are regularly studied and are orthogonal to the branch point base pairs coordination numbers, e.g. J_{twist} and J_{roll}.[22,23] Our 1 μs WT-MetaD-HREXs might not be sufficient to consider the converged sampling of other CVs, which could result in deviated entropy in the free energy calculation. In any cases, we still suggest that our results represent the general free-energy landscapes in the coordination number CV space with only minor deviation, which is supported by the sampled transitions in most of the continuous trajectories in WT-MetaD-HREXs-EXT.

Pure Hamiltonian Replica Exchange and WT-MetaDynamics simulations

We also initially tried the pure HREX as well as WT-MetaDynamics (WT-MetaD) methodologies for our simulations. However, neither of them led to convergence which was the reason why we finally applied the sophisticated combined WT-MetaD-HREX protocol (see the main text).

Regarding the pure HREX, we ran two independent simulations starting from open and closed isomer I states of J1 using the same λs ladder for the 6 replicas and the same replica exchange protocol as we did in subsequent WT-MetaD-HREX. Each HREX was run for 1 μs. As shown in Figure S13, the individual trajectories swiftly separated into two groups which rarely or no longer at all exchanged across the replica ladder. This started right after first few opening/closing transitions which then led to the closed and open HJs permanently occupying the lower and higher replicas, respectively. In the HREX started from closed state isomer I, this happened around 200 ns when two trajectories showing open state HJs almost permanently occupied replicas 5 and 6. Similarly, the HREX started from the open HJ showed one closing event (to isomer I) at about 50 ns with the closed HJ subsequently occupying the replica 0 for the rest of the simulation. We attribute this phenomenon to the relatively large potential energy gaps between the two states, leading to permanent separation between replicas as no further exchange attempts were accepted. This resulted in almost no round trips for the continuous trajectories.

Likewise, the pure WT-MetaD simulations were set up with the same protocol we later used for the WT-MetaD-HREX in the isomer I-open substates space (see main text Methods). The pure WT-MetaD simulations included only a single replica and the scaling protocol we designed for increasing the opening-closing transitions was not applied. Two independent WT-MetaD runs were done for 1 μs each and the free energy profiles obtained. Unlike the WT-MetaD-HREX, the two WT-MetaD runs led to significantly different free energy profiles (Figure S14). In particular, the second run indicated the open HJ state to be more stable than the closed isomer I by ~12.8 kcal/mol, completely contradicting the conclusion we obtained from the standard simulations and the converged WT-MetaD-HREXs. The first WT-MetaD run showed the closed isomer I is more stable than open state by 4.1 kcal/mol, closer to the results of the WT-MetaD-HREX simulations in the same CV space. We also note the statistical errors estimated by our bootstrapping protocol are far lower than the difference of $\Delta G_{\text{isomer I-open}}$ from the two individual WT-MetaD runs. This indicates the pure WT-MetaD protocol was incapable of efficiently sampling the highly complex conformational space of HJ opening-closing transitions, leading to the lack of convergence. Nevertheless, it is noteworthy the WT-MetaD still identified the local free energy minima corresponding to the two half-closed intermediates (Figure S14A, left), confirming these intermediates are not artificially produced by our scaling protocol. In other words, same HJ opening-closing pathways (main text Figure 4) were observed when utilizing CV-based or scaling-based methodologies (or their combination), suggesting that neither of them is biasing the results and that the half-closed intermediates could be realistic.

Side-effects observed in simulations of HJ with short helical arms

In order to reduce computational demands, we also attempted simulations of the J1 system in which we shortened the length of its helical arms to three base pairs each (so called "short HJ", system "J1/short/LMCU/λ0.75"). Although the size reduction of the junction indeed led to significantly faster computational speed compared to larger HJ systems, we observed side effects in form of the spurious inter-helical interactions (Figure S3). These unexpected contacts stabilized the closed state while simultaneously promoting the occurrence of the parallel HJ conformation which is not relevant for our study of the opening-closing transitions. To avoid both issues, we strongly suggest that the length of each HJ arm in simulations should always be at least six and ideally as many as eight base pairs.

Supporting Tables

Table S1. *List of standard MD simulations.*

Simulation name[a]	Number of simulations × Length [μs] (starting structure)[b]
J1/JC[c]	2 × 1 (48nt-closed0)
J1/LM[c]	2 × 1 (48nt-closed0)
J1/JC+CU[c]	2 × 1 (48nt-closed0)
J1/LM/λ0.5	2 × 1 (48nt-closed0) + 2 × 1 (48nt-open0)
J1/LM/λ0.6	1 × 1 + 1 × 2 (48nt-closed0) + 2 × 1 (48nt-open0)
J1/LM/λ0.625	2 × 1 (48nt-closed0)
J1/LM/λ0.65	2 × 1 (48nt-closed0)
J1/LM/λ0.675	2 × 1 (48nt-closed0)
J1/LM/λ0.7	2 × 1 (48nt-closed0) + 2 × 1 (48nt-open0) + 2 × 1 (48nt-open2)
J1/LM/λ0.725	2 × 1 (48nt-closed0) + 2 × 1 (48nt-open0) + 2 × 1 (48nt-open2)
J1/LM/λ0.75	2 × 1 (48nt-closed0) + 2 × 1 (48nt-open0) + 2 × 1 (48nt-open2)
J1/LM/λ0.8	2 × 1 (48nt-closed0) + 2 × 1 (48nt-open0) + 2 × 1 (48nt-open2)
J1/LMCU/λ0.5	2 × 1 (48nt-closed0)
J1/LMCU/λ0.6	2 × 1 (48nt-closed0) + 2 × 2 (48nt-open0)
J1/LMCU/λ0.7	2 × 1 + 1 × 5 (48nt-closed0) + 2 × 1 (48nt-open0) + 2 × 2 (48nt-open2)
J1/LMCU/λ0.725	2 × 1 (48nt-closed0) + 2 × 1 (48nt-closed1) + 2 × 1 (48nt-open0) + 2 × 1 (48nt-open1) + 2 × 2 (48nt-open2)
J1/LMCU/λ0.75	2 × 1 (48nt-closed0) + 2 × 1 + 1 × 10 (48nt-closed1) + 2 × 1 + 1 × 10 (48nt-open1) + 2 × 2 (48nt-open2)
J1/LMCU/λ0.775	2 × 1 (48nt-closed0) + 2 × 1 (48nt-open0) + 2 × 2 (48nt-open2)
J1/LMCU/λ0.8	2 × 1 + 1 × 5 (48nt-closed0) + 2 × 1 (48nt-open0) + 2 × 2 (48nt-open2)
J1/LMCU/λ0.9	2 × 1 + 1 × 5 (48nt-closed0)
J1/LM/15K	2 × 1 (48nt-closed0)
J1/LM/05K	2 × 1 (48nt-closed0)
J1/LM/02K	2 × 1 (48nt-closed0) + 3 × 2 (48nt-open0)
J1/LMCU/02K	3 × 2 (48nt-open0) + 3 × 2 (48nt-closed0)
J1/long/LMCU/λ0.75[d]	4 × 1 + 1 × 5 (64nt-closed0) + 4 × 1 + 1 × 10 (64nt-open0)
J1/huge+long/LMCU[d]	2 × 1 (64nt-closed0) + 2 × 1 (64nt-open0)
J1/short/LMCU/λ0.75	1 × 5 (24nt-closed0) + 1 × 5 (24nt-open0)
J2/LMCU	2 × 1 (48nt-closed0) + 2 × 1 (48nt-open0)
J2/LMCU/λ0.9	2 × 1 (48nt-closed0) + 2 × 1 (48nt-open0)
J2/LMCU/λ0.825	2 × 1 (48nt-closed0)
J2/LMCU/λ0.8	2 × 1 (48nt-closed0) + 2 × 1 (48nt-open0)
J2/LMCU/λ0.775	2 × 1 (48nt-closed0) + 2 × 1 (48nt-open0)
J2/LMCU/λ0.75	2 × 1 (48nt-open0)
J2/LMCU/λ0.725	2 × 1 (48nt-open0)
J13/LMCU	2 × 1 (48nt-closed0) + 2 × 1 (48nt-open0)
J13/LMCU/λ0.9	2 × 1 (48nt-closed0) + 2 × 1 (48nt-open0)
J13/LMCU/λ0.8	2 × 1 (48nt-closed0) + 2 × 1 (48nt-open0)
J13/LMCU/λ0.775	2 × 1 (48nt-closed0) + 2 × 1 (48nt-open0)
J13/LMCU/λ0.75	2 × 1 (48nt-closed0) + 2 × 1 (48nt-open0)
J13/LMCU/λ0.725	2 × 1 (48nt-open0)
J13/LMCU/λ0.7	2 × 1 (48nt-open0)
1BNA[e]	2 × 1 (1BNA)
1BNA/HBfix[e]	2 × 1 (1BNA)
1BNA/λ0.7[e]	2 × 1 (1BNA)
1BNA/λ0.7/HBfix[e]	2 × 1 (1BNA)

[a]"J1", "J2", and "J13" indicate which immobile HJ sequence was simulated. "JC" and "LM" stand for Joung&Cheatham[4] and Li&Merz[27,28] ion parameters, respectively. "CU" indicates that the CUfix modification[5] was applied. "xK" indicates simulations with sub-neutralizing ion conditions with the

“x” referring to the specific concentration (e.g. 05K is c(K^+) = 0.05 M, sub-neutralizing conditions). When not specified, standard net-neutral conditions were applied. The systems with scaling of the branch point base pairs are indicated with “λ” followed by the scaling factor applied (e.g. λ0.75 indicates 0.75 scaling).

[b]The starting structures referred in the parentheses are visualized in Figure S4.

[c]HBfix was not used for the branch point base pairs.

[d]“Long” and “huge” indicate that 64-nt HJ structure and significantly larger solvent box with the distance between the HJ and the box boundary at least 58 Å (so that net-neutral c(K^+) = 20 mM) were used, respectively.

[e]“1BNA” system was prepared from PDB structure 1BNA (Dickerson dodecamer) and used as a control to verify possible side effects of the scaling on standard B-DNA structure. The terms “λ0.7” and “HBfix” refer to the systems with scaling (λ=0.7) on the entire B-DNA helix and with the application of HBfix on the terminal base pairing H-bonds, respectively.

Table S2. *Free-energy differences (kcal/mol) and their standard deviations of all HJ states with the open state used as the reference, in all the WT-MetaD-HREX simulations of the isomer I/II – open state spaces. Negative numbers indicate states more stable than the open state. The ideal λ values giving $\Delta G_{isomer\ I/II\text{-}open}$ =0 in all the runs are listed in the brackets.*

Isomer I – open state space, $c(K^+)$ = 230 mM, first run (ideal λ=0.77)			
Replicas (scaling factor)	Isomer I	Half-closed IL	Half-closed IR
Replica 0 (1)	-6.38±0.77	-0.90±0.21	2.52±0.18
Replica 1 (0.919)	-3.82±0.91	0.39±0.18	3.77±0.16
Replica 2 (0.826)	-1.34±0.99	1.46±0.20	4.66±0.15
Replica 3 (0.727)	1.30±1.16	2.63±0.19	5.83±0.15
Replica 4 (0.640)	3.80±1.21	3.91±0.18	7.00±0.13
Replica 5 (0.551)	5.64±1.05	4.67±0.17	7.58±0.14
Isomer I – open state space, $c(K^+)$ = 230 mM, second run (ideal λ=0.82)			
Replicas (scaling factor)	Isomer I	Half-closed IL	Half-closed IR
Replica 0 (1)	-5.41±1.02	1.31±0.16	3.23±0.11
Replica 1 (0.919)	-2.73±0.84	2.88±0.14	4.64±0.09
Replica 2 (0.826)	-0.07±0.70	4.22±0.13	5.77±0.10
Replica 3 (0.727)	2.67±0.89	5.43±0.16	6.96±0.12
Replica 4 (0.640)	5.32±1.28	6.73±0.13	8.14±0.16
Replica 5 (0.551)	7.62±1.48	7.59±0.16	9.18±0.16
Isomer II – open state space, $c(K^+)$ = 230 mM, first run (ideal λ=0.87)			
Replicas (scaling factor)	Isomer II	Half-closed IIL	Half-closed IIR
Replica 0 (1)	-3.56±0.40	3.11±0.16	0.15±0.17
Replica 1 (0.919)	-1.25±0.54	4.15±0.14	1.17±0.13
Replica 2 (0.826)	1.16±0.50	5.15±0.12	2.24±0.15
Replica 3 (0.727)	3.38±0.43	5.88±0.13	3.04±0.12
Replica 4 (0.640)	5.84±0.67	7.24±0.12	4.37±0.12
Replica 5 (0.551)	7.63±0.64	7.99±0.12	5.20±0.10
Isomer II – open state space, $c(K^+)$ = 230 mM, second run (ideal λ=0.93)			
Replicas (scaling factor)	Isomer II	Half-closed IIL	Half-closed IIR
Replica 0 (1)	-2.16±0.64	2.93±0.14	1.27±0.17
Replica 1 (0.919)	0.25±0.83	3.96±0.14	2.53±0.16
Replica 2 (0.826)	2.60±0.88	4.77±0.14	3.50±0.13
Replica 3 (0.727)	5.35±1.11	6.08±0.11	4.94±0.13
Replica 4 (0.640)	7.04±1.07	6.54±0.12	5.56±0.13
Replica 5 (0.551)	9.06±1.20	7.45±0.14	6.59±0.14
Isomer I – open state space, $c(K^+)$ = 1 M (ideal λ=0.68)			
Replicas (scaling factor)	Isomer I	Half-closed IL	Half-closed IR
Replica 0 (1)	-10.17±0.81	-1.10±0.17	0.49±0.14
Replica 1 (0.919)	-7.90±0.95	-0.42±0.20	1.33±0.14
Replica 2 (0.826)	-4.58±1.29	1.23±0.19	3.13±0.16
Replica 3 (0.727)	-1.17±1.45	2.89±0.18	4.88±0.13
Replica 4 (0.640)	1.45±1.64	4.20±0.18	6.06±0.17
Replica 5 (0.551)	4.21±1.93	5.54±0.19	7.29±0.23
Isomer I – open state space, $c(K^+)$ = 150 mM (ideal λ=0.89)			
Replicas (scaling factor)	Isomer I	Half-closed IL	Half-closed IR
Replica 0 (1)	-3.67±0.90	3.50±0.14	3.99±0.18
Replica 1 (0.919)	-1.02±1.12	4.77±0.13	5.33±0.17
Replica 2 (0.826)	1.90±1.27	6.18±0.12	6.77±0.21
Replica 3 (0.727)	5.26±1.52	7.91±0.13	8.65±0.17
Replica 4 (0.640)	7.64±1.53	9.00±0.14	9.83±0.12
Replica 5 (0.551)	10.59±1.98	10.57±0.16	11.62±0.14
Isomer I – open state space, $c(K^+)$ = 90 mM (ideal λ=0.92)			
Replicas (scaling factor)	Isomer I	Half-closed IL	Half-closed IR
Replica 0 (1)	-2.56±1.11	3.30±0.15	4.73±0.14
Replica 1 (0.919)	0.00±1.28	4.67±0.15	5.95±0.14
Replica 2 (0.826)	2.90±1.57	6.01±0.15	7.38±0.13
Replica 3 (0.727)	5.76±1.27	7.41±0.14	8.77±0.12
Replica 4 (0.640)	7.70±1.04	8.11±0.13	9.60±0.14
Replica 5 (0.551)	10.39±1.46	9.77±0.13	11.13±0.15

Table S3. *Occurrence of parallel HJ conformation in the standard MD simulations.*

Systems	Parallel form (in % of frames)
J1/LM/λ0.6	3.9
J1/LM/λ0.65	7.0
J1/LM/λ0.7	16.6
J1/LM/λ0.75	28.3
J1/LM/λ0.8	0.9
J1/LMCU/λ0.7	0.3
J1/LMCU/λ0.725	3.9
J1/LMCU/λ0.75	22.4
J1/LMCU/λ0.775	7.5
J1/LMCU/λ0.8	21.1
J1/long/LMCU/λ0.75	3.5
J1/short/LMCU/λ0.75	50.8
J1/LMCU/02K	10.7
J2/LMCU	21.2
J2/LMCU/λ0.9	23.2
J2/LMCU/λ0.825	1.2
J2/LMCU/λ0.8	29.9
J2/LMCU/λ0.775	2.9
J13/LMCU/λ0.9	11.0
J13/LMCU/λ0.8	4.8
J13/LMCU/λ0.75	16.0
Total	10.28 (25.185 μs out of the 245 μs ensemble)

Table S4. *List of WT-MetaD-HREX simulations of J1 and the sampled CVs space.*

Sampling space	CVs	# of simulations × Length (μs)	c(K^+)
Isomer I – open state	sI-II$^{0.65}$, sIII-IV$^{0.65}$	2 × 1 (64nt-J1 isomer I)	230 mM
Isomer II – open state	sI-IV$^{0.4}$, sII-III$^{0.4}$	2 × 1 (64nt-J1 isomer II)	230 mM
Isomer I – open state	sI-II$^{0.65}$, sIII-IV$^{0.65}$	1 × 1 (64nt-J1 isomer I)	1 M
Isomer I – open state	sI-II$^{0.65}$, sIII-IV$^{0.65}$	1 × 1 (64nt-J1 isomer I)	150 mM
Isomer I – open state	sI-II$^{0.65}$, sIII-IV$^{0.65}$	1 × 1 (64nt-J1 isomer I)	90 mM

Table S5. *Standard deviations of CVs corresponding to each HJ conformation in WT-MetaD-HREX simulations. sI-II, sIII-IV, sI-IV and sII-III are coordination numbers for the corresponding base pair combinations. The stated values include the exponents of the respective CVs. The σ values of the weighted gaussians in WT-MetaD-HREX were determined considering standard deviations of CVs in all substates.*

Conformations[a]	**sI-II$^{0.65}$**	**sIII-IV$^{0.65}$**	**σ value**
Isomer I	0.8512913	0.8628007	
Open	0.9001168	0.5969054	0.8
Half-closed IL (sI-II closed)	1.917353	1.424672	
Half-closed IR (sIII-IV closed)	0.1395364	0.1000673	
	**sI-IV$^{0.4}$**	**sII-III$^{0.4}$**	**σ value**
Isomer II	0.2344054	0.1917422	
Open	0.2204112	0.3089976	0.2
Half-closed IIL (sI-IV closed)	0.5343098	0.2271776	
Half-closed IIR (sII-III closed)	0.8370706	0.6554123	

[a]We define seven conformations of HJ in our study – isomer I, isomer II, open state, and four half-closed states where one helix is stacked and the other open.

Table S6. *The scaling factors λs utilized in the WT-MetaD-HREX and the HREX simulations.*

Replicas	Scaling factors λs
Replica 0	1 (no scaling)
Replica 1	0.919
Replica 2	0.825
Replica 3	0.727
Replica 4	0.640
Replica 5	0.551

Supplementary Figures

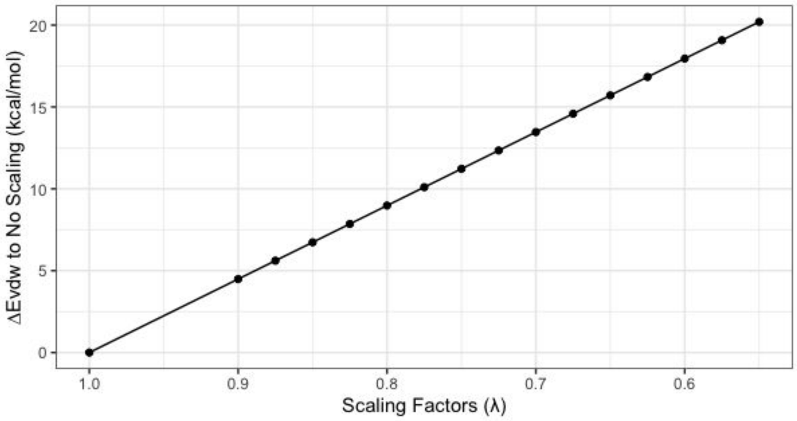

Figure S1. The van der Waals (vdW) energy difference of the branch point nucleotides LJ interaction in the closed state, caused by the scaling with different λs. *The vdW energies were calculated by cpptraj separately for all branch point nucleotides and for nucleotides from either stem I/IV or stem II/III. The LJ interaction energies among the branch point base pairs corresponding to the closed state were then derived by subtracting the latter from the former.*

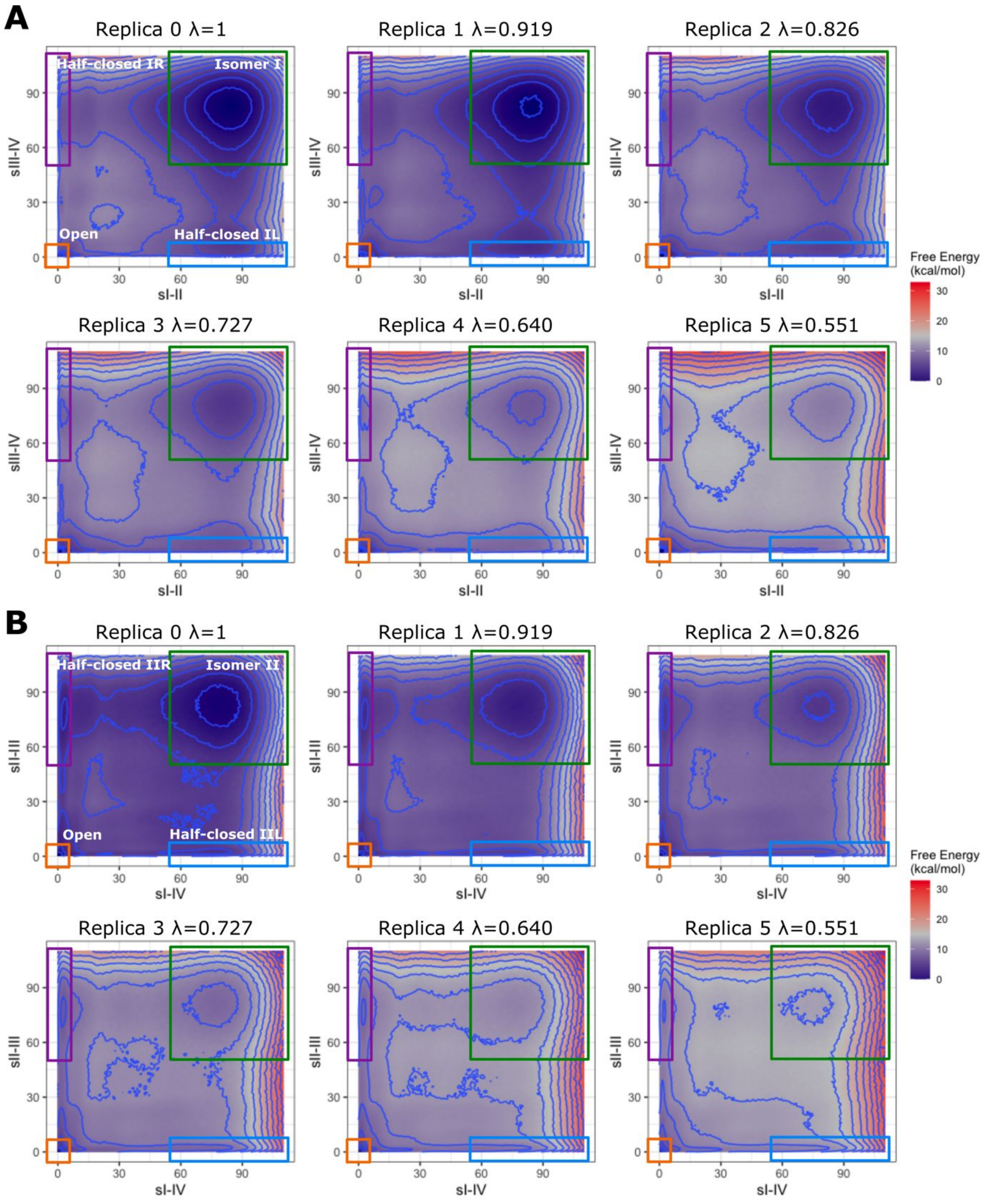

Figure S2. The free-energy profiles of transitions between the open state and the closed states isomer I (A) and II (B) of J1 as shown by the second run of the WT-MetaD-HREX simulations (see Methods for details). *The 2D free-energy profiles are described by the sI-II and sIII-IV (A) and sI-IV and sII-III (B) collective variables (CVs). The four rectangles indicate the approximate regions in the CVs' space corresponding to the individual HJ states (labelled in replicas 0). The interval of the blue contour lines is 2 kcal/mol and the free-energy minimum is set to 0. For the free-energy color gradient, we set 15 kcal/mol as the mid-point (grey). By applying scaling (replicas with larger replica numbers), the open state becomes increasingly preferred over the closed state. Note that the open state is localized in the very small area in bottom left corner, so the free-energy minimum is poorly visible at higher replicas.*

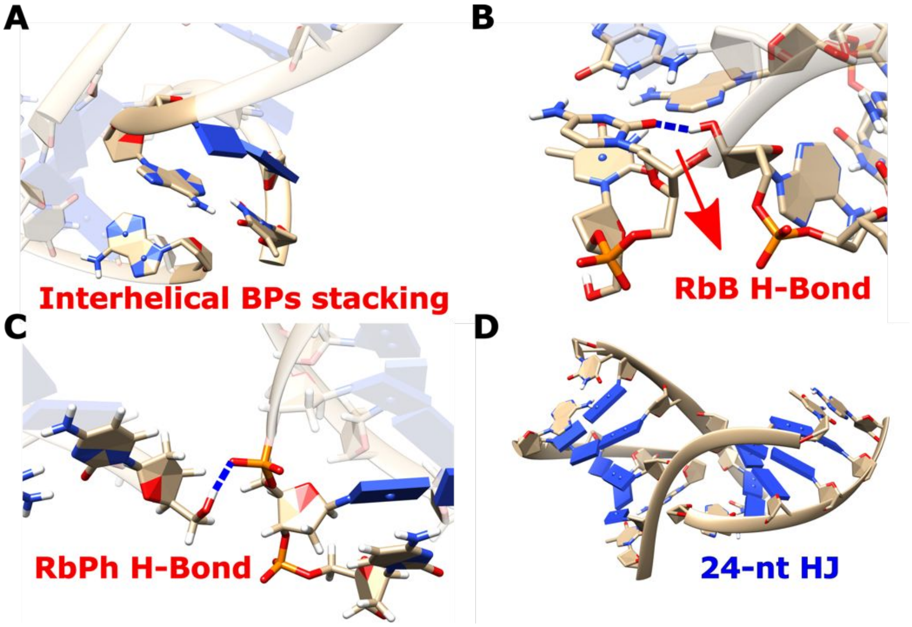

Figure S3. Interhelical interactions commonly observed in MD simulations of the 24-nt HJ (short) systems. *(A) Interhelical base pairs stacking facilitated by terminal base pairs. (B) Interhelical sugar/base and (C) sugar/phosphate H-bond. (D) The 24-nt HJ ("short") structure.*

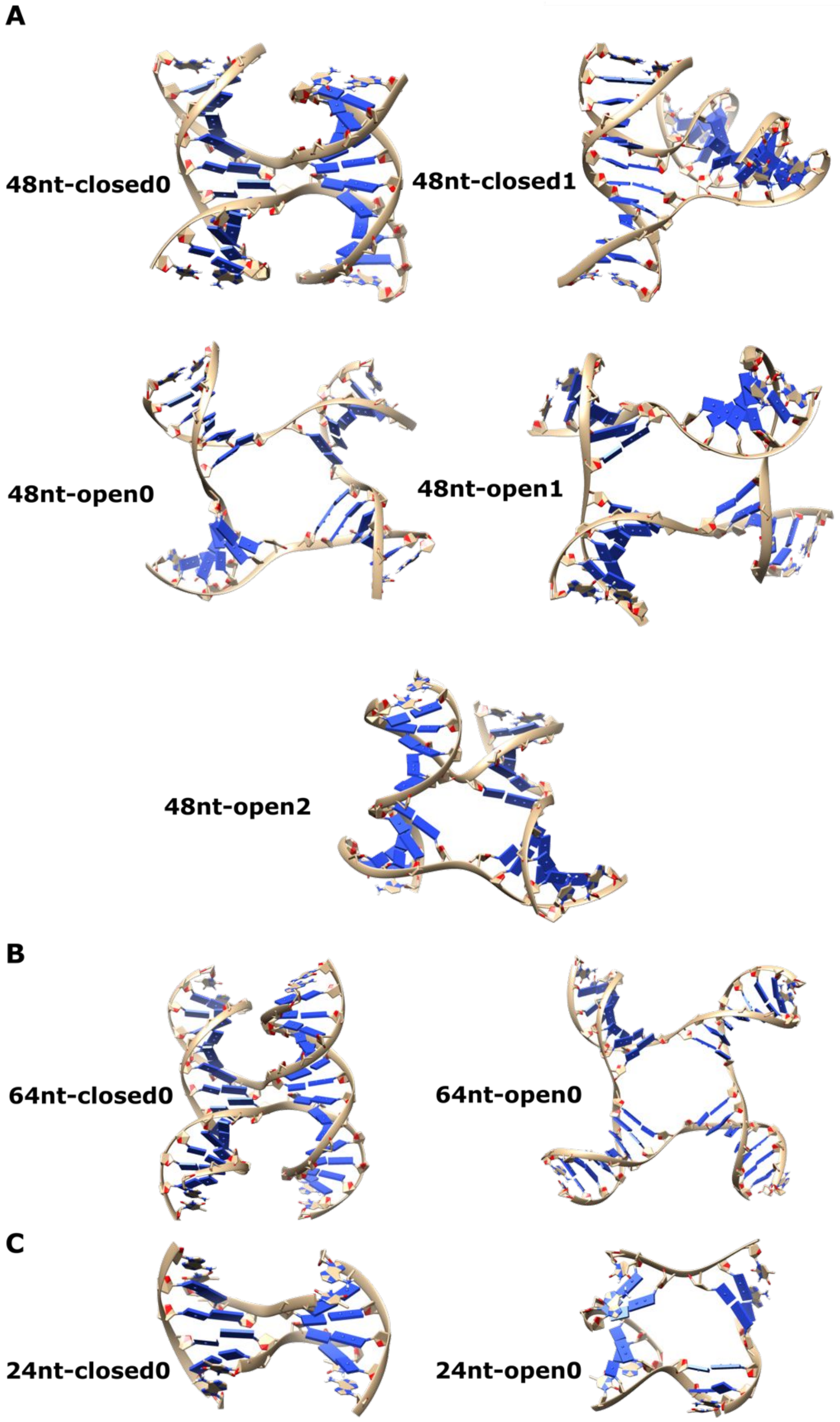

Figure S4. Overview of all starting structures of the J1 junction utilized for MD simulations in this work. *(A) 48-nt J1 structures (B) 64-nt long J1 structures; (C) 24-nt short J1 structures. The closed and open structures of the same type (e.g. 48-nt open0 vs 48nt-open1) possess slightly different geometries. The reason for utilizing multiple starting structures was to increase sampling of the HJ's conformational space in our simulations. The "48nt-closed0" and "48nt-open0" structures were also used to derive starting structures for the simulations of J2 and J13 junctions by mutating the branch point nucleotides.*

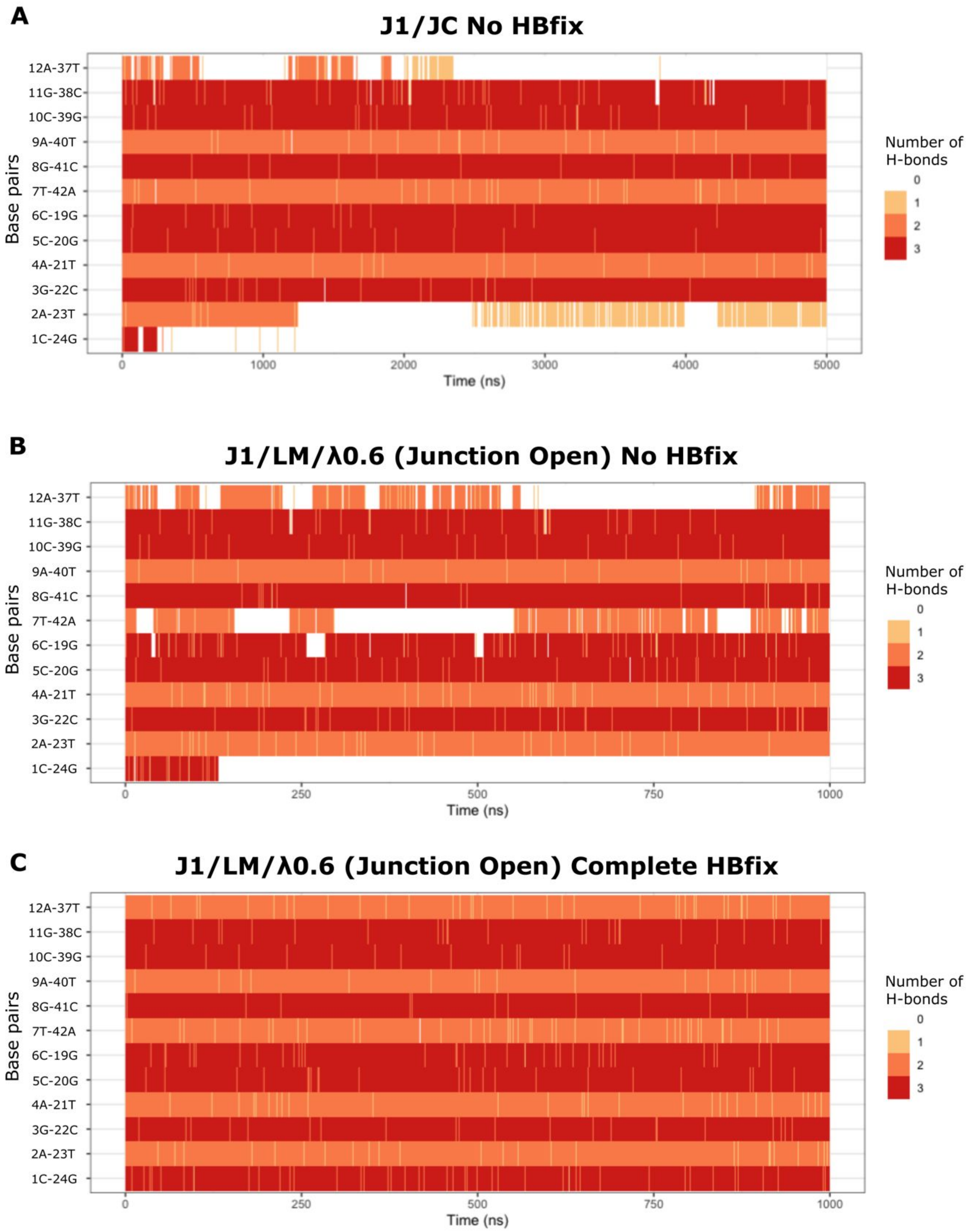

Figure S5. Time development of base-pairing H-bonds in helical arms I and II in selected simulations where we either did or did not use the HBfix to prevent fraying of the terminal base pairs. *The individual base pairs are labelled in the order in which they appear in the continuous strand, from 5' to 3'. (A) J1/JC system which started closed and remained closed; (B, C) J1/LM/λ0.6 systems which started as closed and then opened during the simulation in 50 ns. The "complete HBfix" in (C) indicates the HBfix applied on both the terminal base pairing H-bonds and the branch point base pairing H-bonds.*

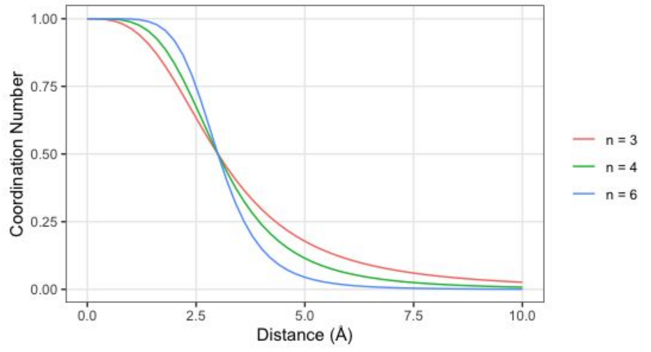

Figure S6. The selection of the n parameter in eq.1 in the main text is based on the distance sensitivity needed for the coordination number. *With higher n value, the slope of the coordination number is steeper around the distance* r_0*. In this study, we used n=4 so that the coordination number function would rapidly approach zero for atomic distance beyond 6 Å.*

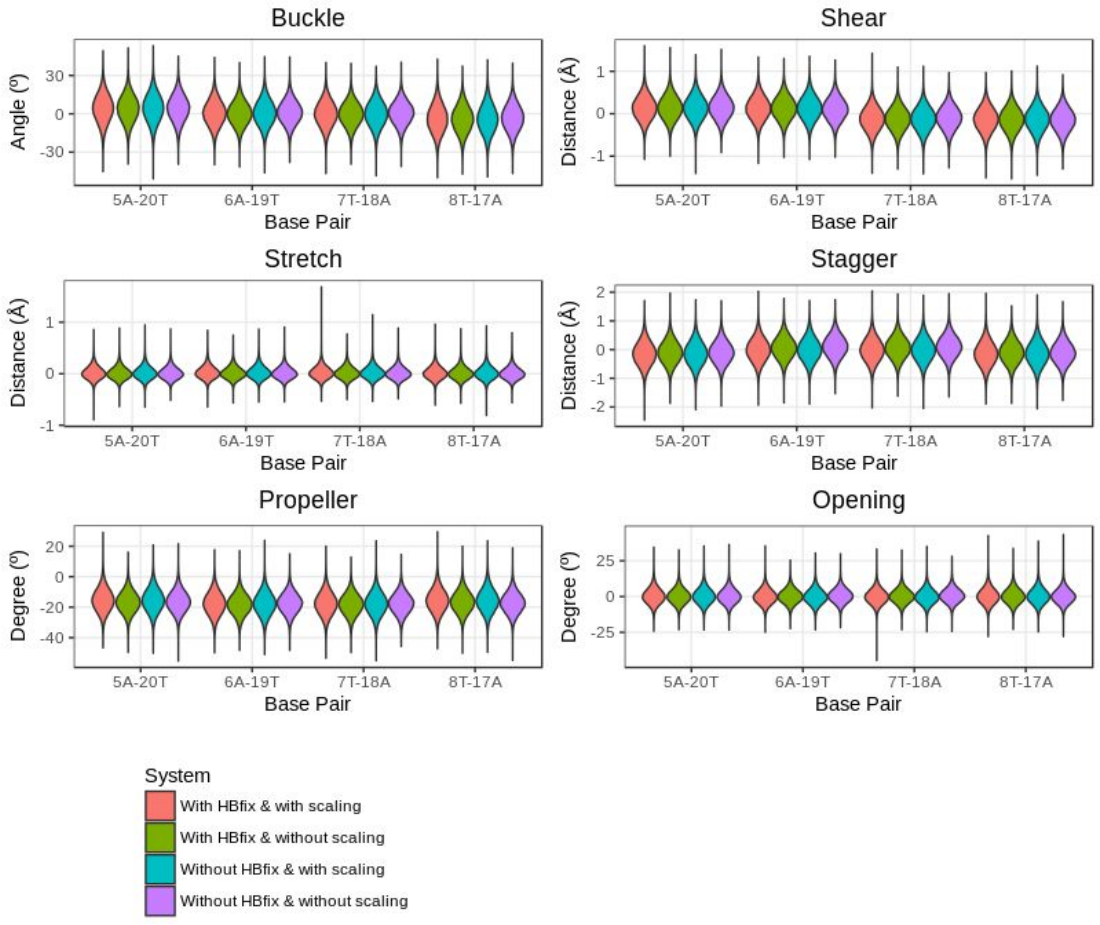

Figure S7. The base pair parameters in B-DNA duplex simulations with no scaling and λ=0.7 scaling applied on all nucleotides, respectively, and with HBfix either applied or not on the H-bonds of terminal base pairing, respectively. *The same scaling scheme was used as for the HJ. The analyzed parameters show no significant difference in their value or distribution in all four systems.*

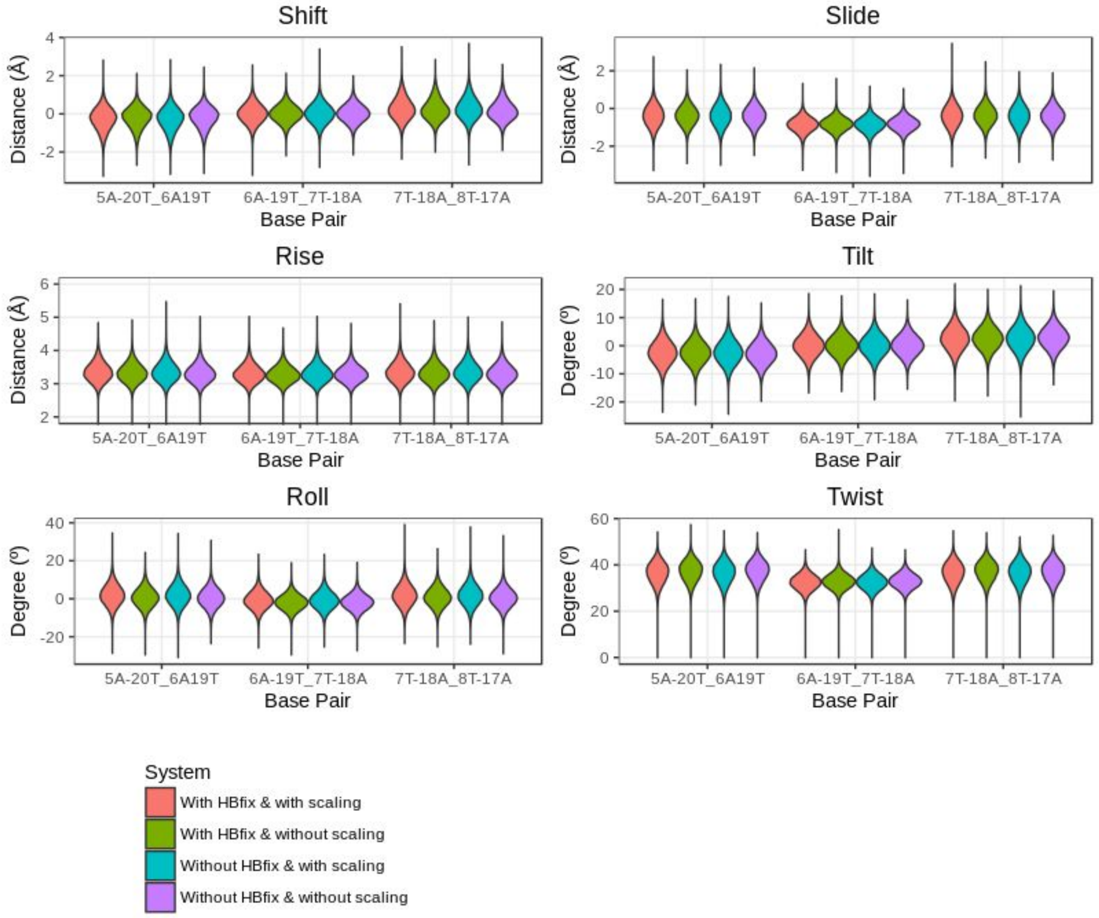

Figure S8. The base pair step parameters in B-DNA duplex simulations. *See the caption of Figure S7.*

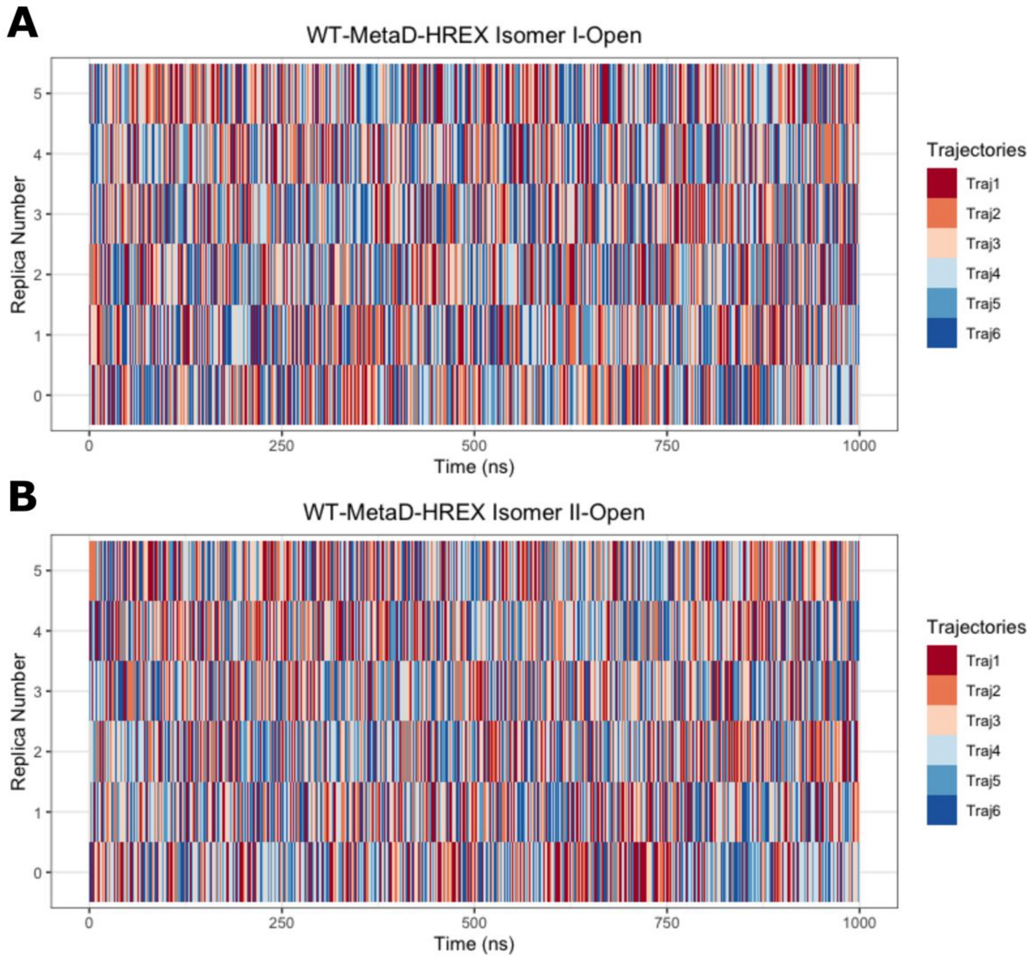

Figure S9. *Time development of the* ***position of each continuous trajectory along the replica ladder in the two WT-MetaD-HREX simulations (the first runs), exploring the isomer I-open (A) or isomer II-open (B) transitions.*** *The continuous trajectories regularly visited all the replicas throughout the simulations.*

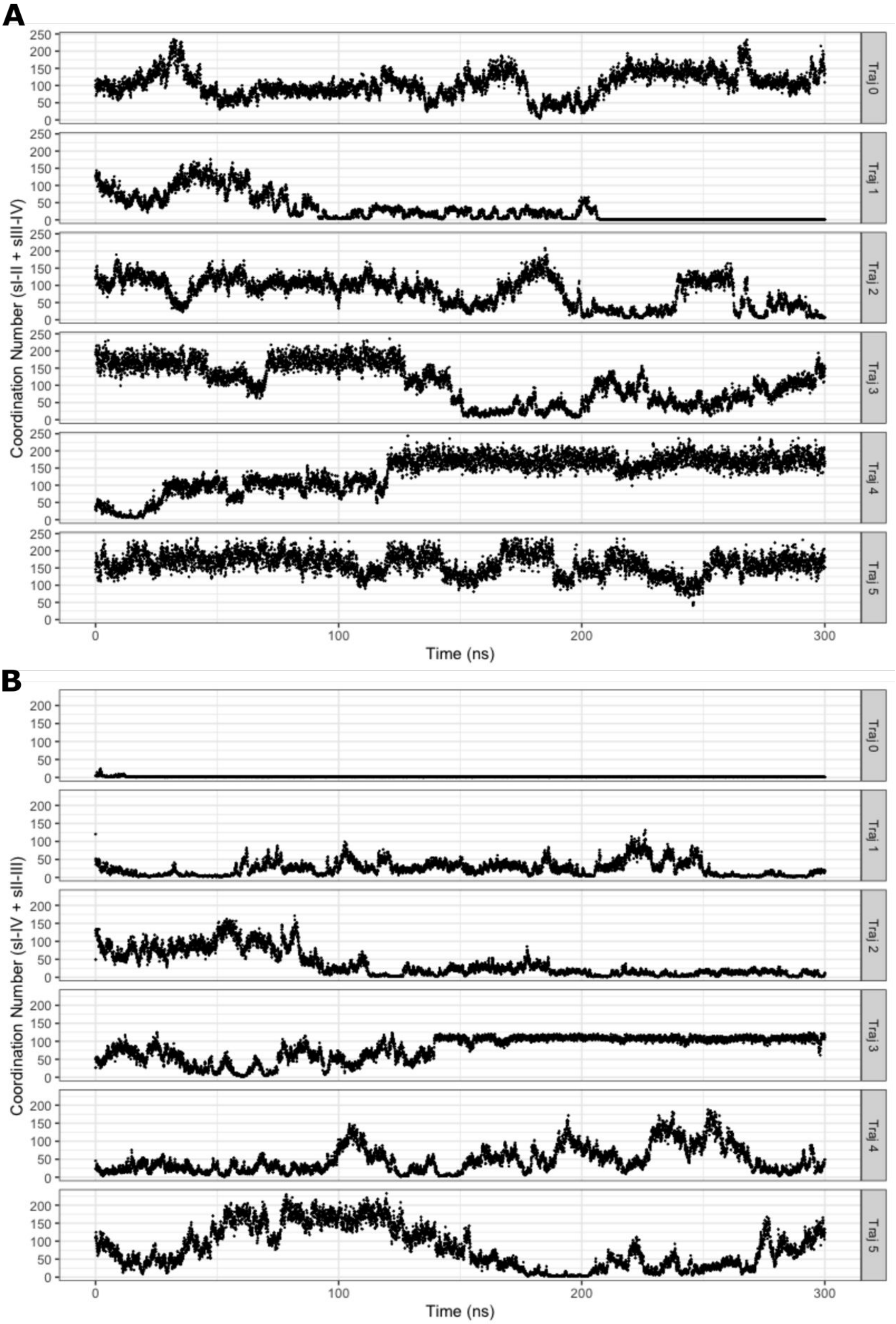

Figure S10. Time-development of the coordination number in the WT-MetaD-HREXs-EXT simulations without new biases added. *With few exceptions, the HJ diffused freely between open and closed states. (A) First run of simulations in isomer I-open sampling space with the coordination number as a sum of sI-II and sIII-IV CVs. (B) First run of simulations in isomer II-open sampling space with the coordination number as a sum of sI-IV and sII-III CVs.*

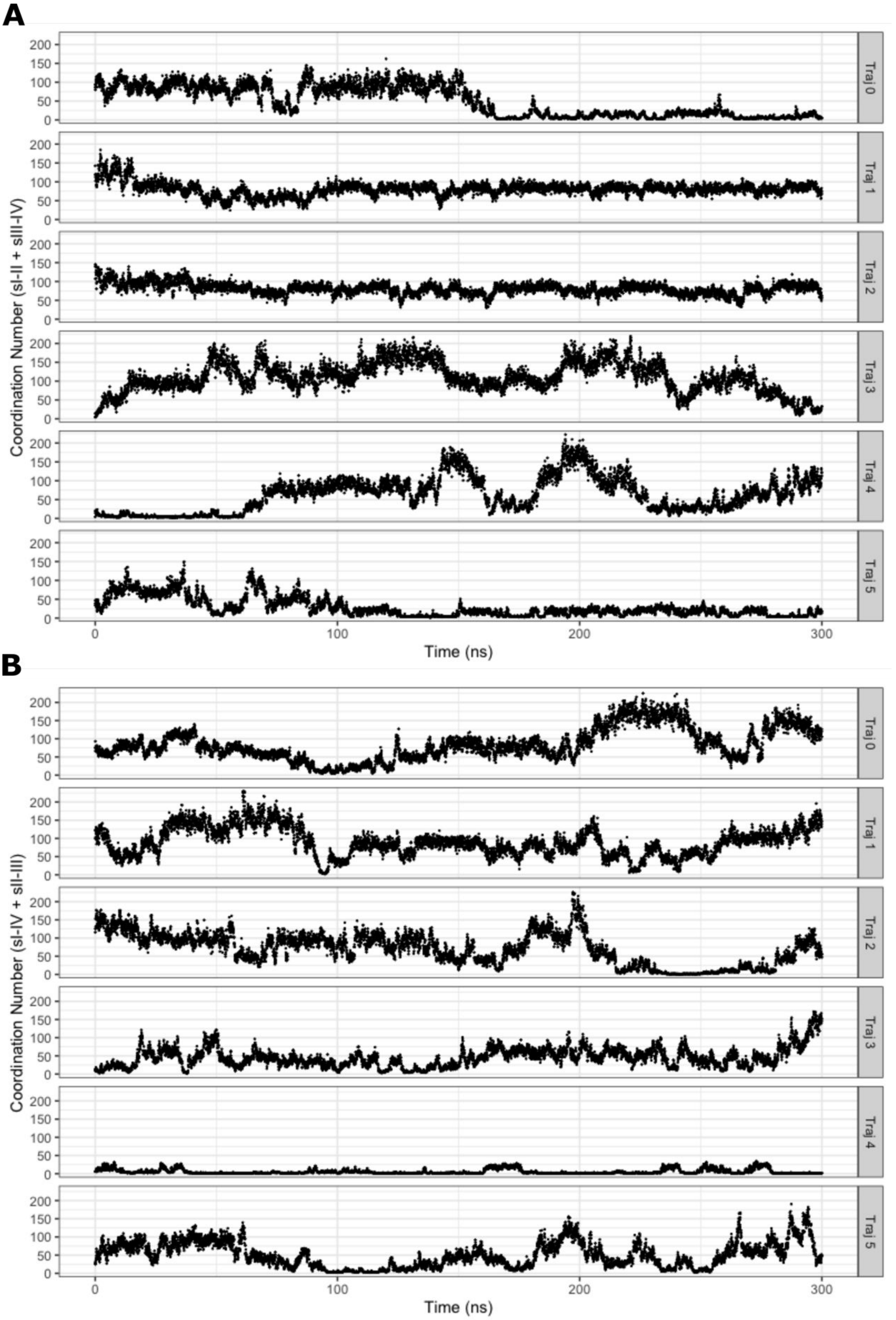

Figure S11. Time-development of the coordination number in the WT-MetaD-HREXs-EXT simulations without new biases added. *With few exceptions, the HJ diffused freely between open and closed states. (A) Second run of simulations in isomer I-open sampling space with coordination number as a sum of sI-II and sIII-IV. (B) Second run of simulations in isomer II-open sampling space with the coordination number as a sum of sI-IV and sII-III.*

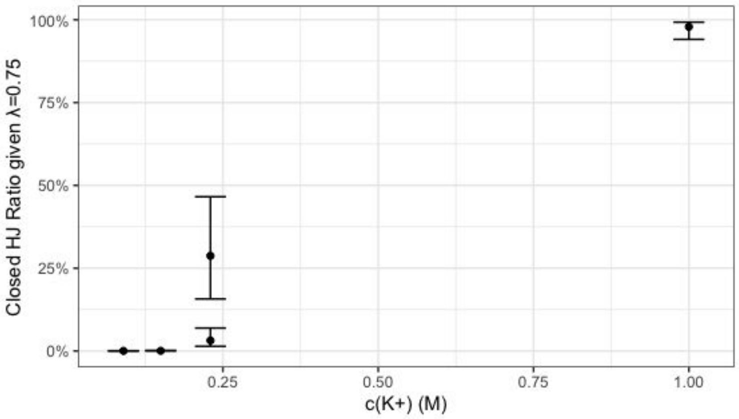

Figure S12. The estimated ratios between open HJ and isomer I at the λ=0.75 scaling value, calculated from the fitting functions in Figure 6 and Figure 8. *The error bars were estimated by bootstrapping analysis. The population ratio shows clear response to the c(K^+). However, note that the c(K^+) in the WT-MetaD-HREX simulations does not directly represent the corresponding bulk concentration one would measure experimentally.*

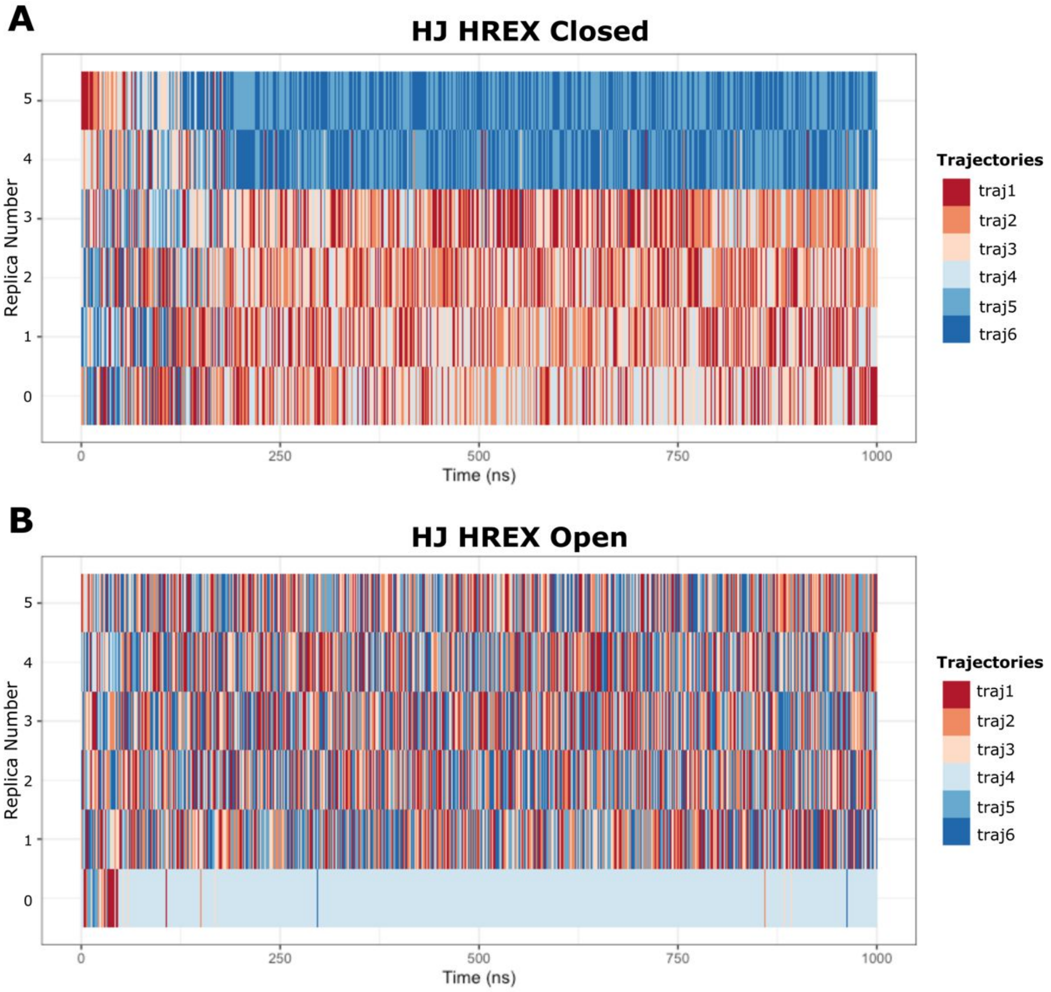

Figure S13. Position of each continuous trajectory along the replica ladder in the two HREX simulations started from isomer I (A) or open (B) state. *The replica spaces were permanently*

separated into two groups early in the simulations, with open and isomer I states populated in higher and lower replicas, respectively, and with virtually no exchanges between the two groups. The method is thus not robust enough to sample the HJ system.

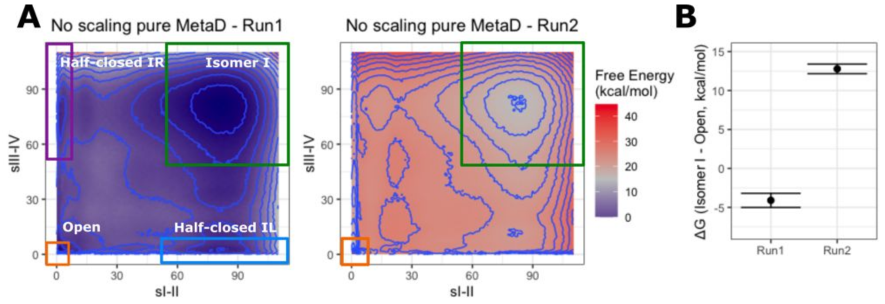

Figure S14. The free-energy profiles of transitions between the open state and the closed state isomer I (A) and the free energy differences between isomer I and the open state (B) in the two pure MetaDynamics runs of J1 without applying the scaling protocol. *(A) The 2D free-energy profiles are described by the sI-II and sIII-IV collective variables (CVs). The four rectangles indicate the approximate regions in the CVs' space corresponding to the individual HJ states (labelled in the first run). The interval of the blue contour lines is 2 kcal/mol and the free-energy minimum is set to 0. For the free-energy color gradient, we set 15 kcal/mol as the mid-point (grey). Note that the open state, localized in the very small area in bottom left corner, is highly preferred in the second run so the free-energy minimum is poorly visible there. In addition, due to the bad convergence of the pure MetaDynamics protocol, the second run shows completely different profile and very unclear free energy minima of the two half-closed states. Therefore, we did not highlight the corresponding areas by rectangles. (B) The free energy differences between the closed isomer I and open states were derived by integrating the probability density in regions defined by (A), with error bars calculated by bootstrapping analysis.*

References:

(1) Case, D. A.; Ben-Shalom, I. Y.; Brozell, S. R.; Cerutti, D. S.; Cheatham, T. E., III; Cruzeiro, V. W. D.; Darden, T. A.; Duke, R. E.; Ghoreishi, D.; Gilson, M. K.; Gohlke, H.; Goetz, A. W.; Greene, D.; Harris, R.; Homeyer, N.; Huang, Y.; Izadi, S.; Kovalenko, A.; Kurtzman, T.; Lee, T. S.; LeGrand, S.; Li, P.; Lin, C.; Liu, J.; Luchko, T.; Luo, R.; Mermelstein, D.; Merz, K. M.; Miao, Y.; Monard, G.; Nguyen, C.; Nguyen, H.; Omelyan, I.; Onufriev, A.; Pan, F.; Qi, R.; Roe, D. R.; Roitberg, A.; Sagui, C.; Schott-Verdugo, S.; Shen, J.; Simmerling, C. L.; Smith, J.; Salomon-Ferrer, R.; Swails, J.; Walker, R. C.; Wang, J.; Wei, H.; Wolf, R. M.; Wu, X.; Xiao, L.; York, D. M.; Kollman, P. A. *Amber 2018*; University of California: San Francisco., 2018.

(2) M.J. Abraham, D. van der Spoel, E. Lindahl, B. H. *GROMACS User Manual Version 2018*; 2018. www.gromacs.org.

(3) Panteva, M. T.; Giambaşu, G. M.; York, D. M. Force Field for Mg 2+ , Mn 2+ , Zn 2+ and Cd 2+ Ions That Have Balanced Interactions with Nucleic Acids HHS Public Access. *J Phys Chem B* **2015**, *119* (50), 15460–15470. https://doi.org/10.1021/acs.jpcb.5b10423.

(4) Joung, I. S.; Cheatham, T. E. Determination of Alkali and Halide Monovalent Ion Parameters for Use in Explicitly Solvated Biomolecular Simulations. *J. Phys. Chem. B.* **2008**, *112* (30), 9020–9041. https://doi.org/10.1021/jp8001614.

(5) Yoo, J.; Aksimentiev, A. New Tricks for Old Dogs: Improving the Accuracy of Biomolecular Force Fields by Pair-Specific Corrections to Non-Bonded Interactions. *Phys. Chem. Chem. Phys.* **2018**, *20* (13), 8432–8449. https://doi.org/10.1039/c7cp08185e.

(6) Steinbrecher, T.; Latzer, J.; Case, D. A. Revised AMBER Parameters for Bioorganic Phosphates. *J Chem Theory Comput.* **2012**, *8* (11), 4405–4412. https://doi.org/10.1021/ct300613v.

(7) Hopkins, C. W.; Le Grand, S.; Walker, R. C.; Roitberg, A. E. Long-Time-Step Molecular Dynamics through Hydrogen Mass Repartitioning. *J. Chem. Theory Comput.* **2015**, *11* (4), 1864–1874. https://doi.org/10.1021/ct5010406.

(8) Kührova, P.; Best, R. B.; Bottaro, S.; Bussi, G.; Šponer, J.; Otyepka, M.; Banáš, P. Computer Folding of RNA Tetraloops: Identification of Key Force Field Deficiencies. *J. Chem. Theory Comput* **2016**, *12* (9), 4534−4548. https://doi.org/10.1021/acs.jctc.6b00300.

(9) Mlýnsky, V.; Kührova, P.; Kühr, T.; Otyepka, M.; Bussi, G.; Banáš, P.; Šponer, J. Fine-Tuning of the AMBER RNA Force Field with a New Term Adjusting Interactions of Terminal Nucleotides. *J. Chem. Theory Comput* **2020**, *16* (6), 3936–3946. https://doi.org/10.1021/acs.jctc.0c00228.

(10) Krepl, M.; Pokorna, P.; Mlýnský, V.; Stadlbauer, P.; Šponer, J. Spontaneous Binding of Single-Stranded RNAs to RRM Proteins Visualised by Unbiased Atomistic Simulations with Rescaled RNA Force Field. *Nucleic Acids Res.* **2022**, *50* (21), 12480–12496. https://doi.org/10.1093/nar/gkac1106.

(11) Drew, H. R.; Wing, R. M.; Takano, T.; Broka, C.; Tanaka, S.; Itakura, K.; Dickerson, R. E. Structure of a B-DNA Dodecamer: Conformation and Dynamics. *Proc. Natl. Acad. Sci. U.S.A.* **1981**, *78* (4), 2179–2183. https://doi.org/10.1073/pnas.78.4.2179.

(12) Lu, X.-J.; Olson, W. K. 3DNA: A Software Package for the Analysis, Rebuilding and Visualization of Three-Dimensional Nucleic Acid Structures. *Nucleic Acids Res.* **2003**, *31* (17), 5108–5121. https://doi.org/10.1093/nar/gkg680.

(13) Tribello, G. A.; Bonomi, M.; Branduardi, D.; Camilloni, C.; Bussi, G. PLUMED 2: New Feathers for an Old Bird. *Comput. Phys. Commun.* **2014**, *185* (2), 604–613. https://doi.org/10.1016/j.cpc.2013.09.018.

(14) Bonomi, M.; Branduardi, D.; Bussi, G.; Camilloni, C.; Provasi, D.; Raiteri, P.; Donadio, D.; Marinelli, F.; Pietrucci, F.; Broglia, R. A.; Parrinello, M. PLUMED: A Portable Plugin for Free-Energy Calculations with Molecular Dynamics. *Comput. Phys. Commun.* **2009**, *180* (10), 1961–1972. https://doi.org/10.1016/j.cpc.2009.05.011.

(15) Grubmüller, H.; Heymann, B.; Tavan, P. Ligand Binding: Molecular Mechanics Calculation of the Streptavidin-Biotin Rupture Force. *Science (80-.).* **1996**, *271* (5251), 997–999. https://doi.org/10.1126/science.271.5251.997.

(16) Branduardi, D.; Bussi, G.; Parrinello, M. Metadynamics with Adaptive Gaussians. **2012**. https://doi.org/10.1021/ct3002464.

(17) Bussi, G.; Laio, A. Using Metadynamics to Explore Complex Free-Energy Landscapes. *Nat. Rev. Phys.* **2020**, *2* (4), 200–212. https://doi.org/10.1038/s42254-020-0153-0.

(18) Rosenblatt, M. On the Estimation of Regression Coefficients of a Vector-Valued Time Series with a Stationary Residual. *Ann. Math. Stat.* **1956**, *27* (1), 99–121.

(19) Grossfield, A.; Zuckerman, D. M. Quantifying Uncertainty and Sampling Quality in Biomolecular Simulations. *Annu Rep Comput Chem* **2009**, *1* (5), 23–48. https://doi.org/10.1016/S1574-1400(09)00502-7.

(20) Grossfield, A.; Patrone, P. N.; Roe, D. R.; Schultz, A. J.; Siderius, D.; Zuckerman, D. M. Best Practices for Quantification of Uncertainty and Sampling Quality in Molecular Simulations [Article v1.0]. *Living J. Comput. Mol. Sci.* **2019**, *1* (1), 1–24. https://doi.org/10.33011/livecoms.1.1.5067.

(21) Mlýnský, V.; Janeček, M.; Kührová, P.; Fröhlking, T.; Otyepka, M.; Bussi, G.; Banás, P.; Šponer, J. Toward Convergence in Folding Simulations of RNA Tetraloops: Comparison of Enhanced Sampling Techniques and Effects of Force Field Modifications. *J. Chem. Theory Comput.* **2022**, *18* (4), 2642–2656. https://doi.org/10.1021/acs.jctc.1c01222.

(22) Adendorff, M. R.; Tang, G. Q.; Millar, D. P.; Bathe, M.; Bricker, W. P. Computational Investigation of the Impact of Core Sequence on Immobile DNA Four-Way Junction Structure and Dynamics. *Nucleic Acids Res.* **2022**, *50* (2), 717–730. https://doi.org/10.1093/nar/gkab1246.

(23) Watson, J.; Hays, F. A.; Ho, P. S. Definitions and Analysis of DNA Holliday Junction Geometry. *Nucleic Acids Res.* **2004**, *32* (10), 3017–3027. https://doi.org/10.1093/nar/gkh631.

(24) Chen, A. A.; Draper, D. E.; Pappu, R. V. Molecular Simulation Studies of Monovalent Counterion-Mediated Interactions in a Model RNA Kissing Loop. *J. Mol. Biol.* **2009**, *390* (4), 805–819. https://doi.org/10.1016/j.jmb.2009.05.071.

(25) Šponer, J.; Bussi, G.; Krepl, M.; Banáš, P.; Bottaro, S.; Cunha, R. A.; Gil-Ley, A.; Pinamonti, G.; Poblete, S.; Jurečka, P.; Walter, N. G.; Otyepka, M. RNA Structural Dynamics as Captured by Molecular Simulations: A Comprehensive Overview. *Chem. Rev.* **2018**, *118* (8), 4177–4338. https://doi.org/10.1021/acs.chemrev.7b00427.

(26) Hub, J. S.; De Groot, B. L.; Grubmü, H.; Groenhof, G. Quantifying Artifacts in Ewald Simulations of Inhomogeneous Systems with a Net Charge. **2013**. https://doi.org/10.1021/ct400626b.

(27) Li, P.; Song, L. F.; Merz, K. M. Systematic Parameterization of Monovalent Ions Employing the Nonbonded Model. *J. Chem. Theory Comput.* **2015**, *11* (4), 1645–1657. https://doi.org/10.1021/ct500918t.

(28) Li, P.; Song, L. F.; Merz, K. M. Parameterization of Highly Charged Metal Ions Using the 12-6-4 LJ-Type Nonbonded Model in Explicit Water. *J. Phys. Chem. B* **2015**, *119* (3), 883–895. https://doi.org/10.1021/jp505875v.
